# Artificial superconducting Kondo lattice in a van der Waals heterostructure

**DOI:** 10.1038/s41467-024-53166-9

**Published:** 2024-10-11

**Authors:** Kai Fan, Heng Jin, Bing Huang, Guijing Duan, Rong Yu, Zhen-Yu Liu, Hui-Nan Xia, Li-Si Liu, Yao Zhang, Tao Xie, Qiao-Yin Tang, Gang Chen, Wen-Hao Zhang, F. C. Chen, X. Luo, W. J. Lu, Y. P. Sun, Ying-Shuang Fu

**Affiliations:** 1 School of Physics and Wuhan National High Magnetic Field Center, Huazhong University of Science and Technology, Wuhan, 430074 China; 2 School of Physics and Astronomy, Beijing Normal University, Beijing, 100875 China; 3 Beijing Computational Science Research Center, Beijing, 100093 China; 4 Department of Physics and Beijing Key Laboratory of Opto-electronic Functional Materials and Micro-nano Devices, Renmin University of China, Beijing, 100872 China; 5 Key Laboratory of Materials Physics, Institute of Solid State Physics, Chinese Academy of Sciences, Hefei, 230031 China; 6 High Magnetic Field Laboratory, Chinese Academy of Sciences, Hefei, 230031 China; 7 Collaborative Innovation Center of Advanced Microstructures, Nanjing University, Nanjing, 210093 China; 8 Wuhan Institute of Quantum Technology, Wuhan, 430206 China

**Keywords:** Electronic properties and materials, Magnetic properties and materials, Superconducting properties and materials

## Abstract

Engineering Kondo lattice with tailored functionality is desirable for elucidating the heavy fermion physics. We realize the construction of an artificial Kondo lattice/superconductor heterojunction by growing monolayer VSe_2_ on bulk 2H-NbSe_2_ with molecular beam epitaxy. Spectroscopic imaging scanning tunneling microscopy measurements show the emergence of a new charge density wave (CDW) phase with $\sqrt{3}\times$ $\sqrt{3}$ periodicity on the monolayer VSe_2_. Unexpectedly, a pronounced Kondo resonance appears around the Fermi level, and distributes uniformly over the entire film, evidencing the formation of Kondo lattice. Density functional theory calculations suggest the existence of magnetic interstitial V atoms in VSe_2_/NbSe_2_, which play a key role in forming the CDW phase along with the Kondo lattice observed in VSe_2_. The Kondo origin is verified from both the magnetic field and temperature dependences of the resonance peak, yielding a Kondo temperature of ~ 44 K. Moreover, a superconducting proximity gap opens on monolayer VSe_2_, whose shape deviates from the function of one-band BCS superconductor, but is reproduced by model calculations with heavy electrons participating the pairing condensate. Our work lays the experimental foundation for studying interactions between the heavy fermion liquids and the superconducting condensate.

## Abstract (web summary)

The authors observe a coherent Kondo lattice in a monolayer of VSe_2_ grown on a superconducting 2H-NbSe_2_ substrate. Superconductivity is established in the Kondo lattice through proximity effect from the substrate.

## Introduction

Kondo lattice originates from the hybridization between a periodic array of local magnetic moments and conduction electrons, resulting in deconfinement of local moments and concomitant strong enhancement in the effective mass of low energy electrons, which constitutes the basic theoretical model for heavy fermion materials^1–4^. As dictated by the intricate interplay between onsite Kondo screening and inter-site exchange coupling, a wealth of exotic quantum phases occur in Kondo lattice systems that include quantum criticality^5–8^, non-Fermi liquid behavior^9–12^, and unconventional superconductivity^13–16^.

Conventional heavy fermion materials are f-electron compounds. Recent advances in the research of van der Waals (vdW) materials have revealed that heavy fermions can also exist in d-electron systems, which contribute to a deeper understanding of Kondo lattice physics^17–22^. Based on the more extended nature of d-electrons and their designability, vdW materials provide rich opportunities for artificially tailoring the Kondo lattice systems with desired functionalities^23–25^. One notable example of such kind is the superconducting (SC) proximity effect, which has shown its success in enforcing superfluid condensate in nanostructures with strong spin-orbit coupling for realizing topological superconductivity^26^. It would be a priori to expect SC pairing could also be induced in Kondo lattice via SC proximity effect. But the mechanism of superconductivity, in particular the role of heavy electrons, is still unclear. Despite its theoretical interest^27–30^, the experimental system hosting such a proximitized SC Kondo lattice is yet realized.

In this study, our spectroscopic imaging scanning tunneling microscopy (SI-STM) measurements on the monolayer VSe_2_ unveil a new ($\sqrt{3}\times \sqrt{3}$) charge density wave (CDW) phase and a pronounced Kondo resonance, as is evidenced from its temperature and magnetic field dependences. The Kondo resonance distributes uniformly over the entire film, signifying the formation of a Kondo lattice with delocalization of the local moments. Density functional theory (DFT) calculations indicate the existence of interstitial V atoms between VSe_2_/NbSe_2_, which play a key role in generating the Kondo effect. Moreover, a proximitized SC gap emerges on monolayer VSe_2_, whose spectral shape cannot be perfectly fitted by the simple one-band BCS function^31–33^, but is reproduced by our Kondo lattice model involving heavy electrons in the SC pairing. Our study not only enriches the artificial heavy fermion system from vdW materials with d-electrons, but also provides a platform for in-depth investigation of the interplay between superconductivity and heavy electron fluids.

The experiments are performed with a custom-made cryogenic Unisoku STM system^34^. High-quality monolayer VSe_2_ films are grown by molecular beam epitaxy on a cleaved 2H-NbSe_2_ substrate. Our first-principles calculations are performed with Vienna ab initio simulation package (VASP)^35^. Detailed descriptions of the experiments and the calculations are depicted in the Supplemental Materials.

## Results

**Fig. 1:**
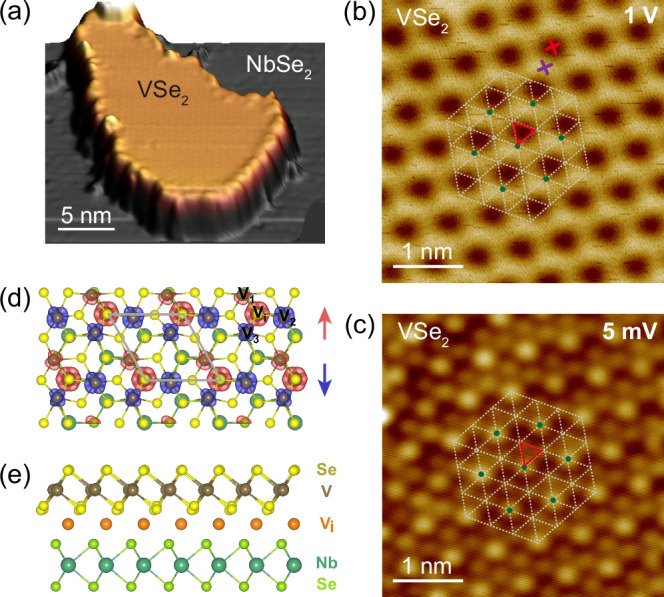
Morphology and atomic structure of √3 × √3 VSe_2_ grown on NbSe_2_. **a** STM topography (*V*_b_ = -1 V, *I*_t_ = 10 pA) of a VSe_2_ island. **b** Magnified STM image (*V*_b_ = 1 V, *I*_t_ = 10 pA) of $\sqrt{3}$ $\times \sqrt{3}$ VSe_2._ The red triangle marks the trimer unit. The white dashed lines and the green dots represent the atomic lattice and the $\sqrt{3}$ $\times \sqrt{3}$ period, respectively. **c** Atomic resolution image of $\sqrt{3}$ $\times \sqrt{3}$ VSe_2_ (*V*_b_ = 5 mV, *I*_t_ = 5 pA). **d** Top view of $\sqrt{3}$ $\times \sqrt{3}$ VSe_2_/NbSe_2_ junction with intercalated V atoms, determined by the DFT calculations. The calculated spin density distribution is illustrated with red for spin-up (↑) and blue for spin-down (↓)_._ The isosurface is set to 0.01 e/Å^3^ for the spin-density plot. The magnetic moments of V_i_, V_1_, V_2_ and V_3_ are 1, 0.2, −0.7 and −0.4 ${\mu }_{B}$ respectively. The $\sqrt{3}\times \sqrt{3}$ superlattice is illustrated with grey solid line. **e** Side view of the heterostructure.

We found the morphology and stoichiometry of the V-Se compounds grown on NbSe_2_ evolve from V_2_Se_9_^36,37^, coexisting $2\times \sqrt{3}$ and $\sqrt{7}\times \sqrt{3}$ CDW phases^38–40^, to $\sqrt{3}\times \sqrt{3}$ CDW phase upon elevating the substrate temperature and the concomitant V:Se flux ratios (Figs. S1, S2). By delicately controlling the growth condition, monolayer VSe_2_ with uniform $\sqrt{3}\times \sqrt{3}$ phase was achieved for the first time. Figure 1a, b shows the STM morphology of the monolayer VSe_2_. Its atomic resolution image acquired at 5 mV resolves the triangular lattice of the top layer Se (Fig. 1c, white dashed lines). The measured in-plane lattice constant of 0.35 nm and the monolayer height of ~0.60 nm both conform to those of the 1T-VSe_2_^41,42^. The atomic resolution image also features an evident $\sqrt{3}\times \sqrt{3}$ superstructure, where one (Fig. 1c, green dot) of every three Se atoms (Fig. 1c, red triangle) becomes brighter than the other two. The STM morphology of the $\sqrt{3}\times \sqrt{3}$ superstructure drastically changes with bias, which appears as a honeycomb at 1 V (Figs. 1b, S6), demonstrating its distinct electronic property.

**Fig. 2:**
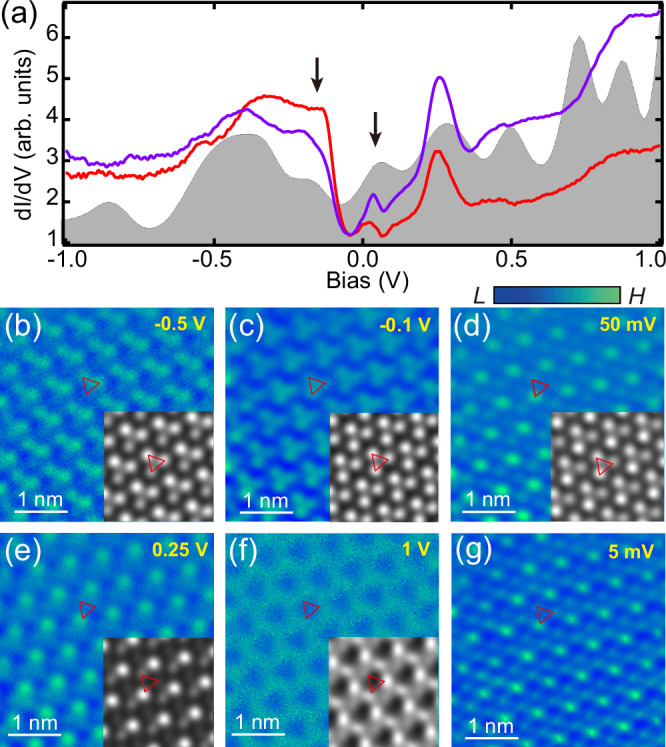
Experimental and DFT-calculated d*I*/d*V* spectra and mappings of √3 × √3 VSe_2_. **a** Typical tunneling spectra of $\sqrt{3}$ $\times \sqrt{3}$ VSe_2_ at locations marked with red and purple crosses in Fig. 1b (*V*_b_ = −1 V, *I*_t_ = 200 pA, *V*_mod_ = 20 mV). The CDW gap edges are marked with black arrows. The shadowed grey curve depicts DFT-calculated local DOS based on the intercalated structure of Fig. 1d, e. **b**–**g** Constant-height d*I*/d*V* mappings of the same area as Fig. 1b at different biases. Inset images show the DFT-simulated d*I*/d*V* mappings at corresponding biases.

Typical tunneling spectra of the VSe_2_ indicate an evident gap of ~155 meV spanning the Fermi surface with gap edges locating at about −130 mV and 25 mV, respectively (Fig. 2a). Those spectroscopic intensities spatially vary (Fig. S4), where the d*I*/d*V* mappings drastically evolve with bias (Figs. 2b–g and S7). Specifically, each unit of the $\sqrt{3}\times \sqrt{3}$ pattern contains a Se atomic trimer of similar intensity (red triangle) at the bias range of about [−0.3 V, −0.1 V] (Fig. 2c). One atom of the Se trimer appears brighter than the other two atoms at low bias of [−0.05 V, 0.3 V] (Fig. 2d, e), but becomes darker below −0.4 V (Fig. 2b). This observation, in conjunction with the growth kinetics and DFT-calculations shown later, suggests the existence of intercalated V atoms in the heterostructure interface (Supplementary Note 1). On the other hand, as shown in Fig. S5, spatial intensities at the two spectral gap edges show a clear anti-phase relationship, which is reminiscent of the CDW phase^43^.

**Fig. 3:**
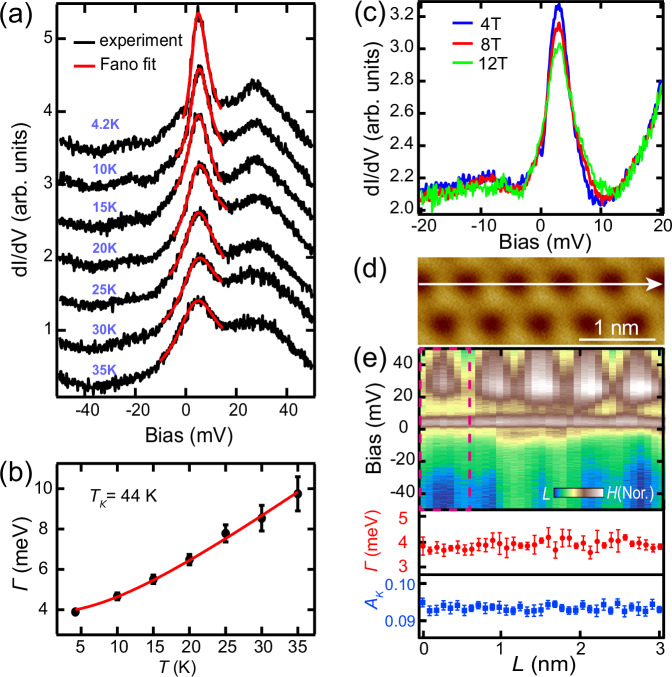
Temperature and magnetic field dependences of the Kondo resonance. **a** Temperature dependence of the Kondo resonance spectra at the same point (*V*_b_ = 50 mV, *I*_t_ = 200 pA, *V*_mod_ = 0.5 mV) and their Fano fittings. The spectra are offset for clarity. **b** Temperature dependence of the Kondo resonance width *Γ* obtained from the Fano fitting from **a**. The red line is a fitting to the data with the Kondo model, giving a Kondo temperature of *T*_*K*_ = 44 K. The error bars are from the Fano fitting. **c** The Kondo resonance spectra (*V*_b_ = 20 mV, *I*_t_ = 200 pA, *V*_mod_ = 0.3 mV) measured under different magnetic fields. **d** STM image of VSe_2_ surface containing multiple units of the $\sqrt{3}\times \sqrt{3}$ pattern (*V*_b_ = 1 V, *I*_t_ = 10 pA). **e** The upper panel is a normalized 2D conductance plot of the Kondo resonance (*V*_b_ = 50 mV, *I*_t_ = 200 pA, *V*_mod_ = 0.5 mV) taken along the white line marked in **d**, whose fitted Kondo resonance width *Γ* and amplitude *A*_*K*_ are shown in the lower panels. Bin size of the 2D conductance plot is 86 pm, which is significantly smaller than the CDW period of 610 pm.

Next, we scrutinize the fine spectroscopic features around *E*_F_ to investigate the low energy excitations. Intriguingly, a pronounced sharp resonance surpassing *E*_F_ emerges, which is peaked at 3 meV and superimposed on a broad peak that is centered at about 25 meV (Fig. 3a, black curve). Its narrow peak width suggests it cannot be from conventional electronic state, but is relevant to many-body spin excitation and the most probable origin is the Kondo resonance^44,45^. As such, we fitted the spectrum with a Fano line shape with a linear background $\frac{{dI}(V)}{{dV}}=A\frac{{(q+\varepsilon )}^{2}}{1+{\varepsilon }^{2}}+B$, where $\varepsilon=\frac{({eV}-{\varepsilon }_{0})}{\varGamma }$, $B=a \cdot V+b$ with the Fano factor *q*, the Kondo resonance energy ${\varepsilon }_{0}$, and the intrinsic Kondo resonance width *Γ*. The fitting nicely matches the experimental spectrum, which gives a Kondo peak width *Γ* of 3.9 meV and a Fano factor *q* of 6 (Fig. 3a, red curve]. In addition, the spectra of islands with different sizes exhibit same Kondo peaks (Fig. S22), ruling out the influence of quantum size effects.

The Kondo resonance is further justified from its temperature and magnetic field evolutions. A rigorous evidence for the Kondo resonance is its temperature evolution. The resonance broadens rapidly with increasing temperature (Fig. 3a, black curves). Fano fittings to the spectra at different temperatures (Fig. 3a, red curves) extract the intrinsic *Γ* (Fig. 3b, black dots) after subtracting the influences from the thermal broadening and the lock-in modulation^46^. The relation of *Γ* as a function of temperature *T* can be excellently fitted using the well-established expression for Kondo systems^45–47^, i.e. $2\varGamma \left({{\rm{T}}}\right)=\sqrt{{(\alpha {k}_{B}T)}^{2}+{(2{k}_{B}{T}_{k})}^{2}}$, which yields a Kondo temperature ${T}_{k}$ ~ 44 K at $\alpha$ = 5.1 (Fig. 3b). Moreover, upon applying magnetic field at 0.6 K, the resonance becomes suppressed with decreased peak height and increased peak width (Fig. 3c), conforming to the expected behavior of a Kondo resonance. A Kondo peak splitting is not observed even under 12 T due to the large peak width.

Spatial distribution of the Kondo resonance is measured with conductance mapping at 5 mV (Fig. 2g), corresponding to the energy of the Kondo state. As the Kondo resonance is superimposed on the spectral background from the CDW pattern, which introduces a periodically modulated electron density pattern in real space. As a result, the Kondo peak intensities from direct spectroscopic measurements also exhibit the same spatial pattern as the CDW. Each CDW unit presumably hosts a localized magnetic moment in a similar fashion as the star of David motifs in 1T-TaS_2_^21^, forming a spin matrix that interacts with itinerant electrons. Line spectra surpassing multiple units of the $\sqrt{3}\times \sqrt{3}$ pattern show that the Kondo resonance prevails over the entire surface (Fig. 3e). The fitted Kondo resonance widths and Kondo amplitudes along the line are reasonably uniform, demonstrating the global nature of the Kondo lattice with the establishment of coherent Kondo screening^48^. To more clearly show the spatial homogeneity of the normalized Kondo resonance, we selected all the spectra from the 2D conductance plot of Fig. 3e within a single CDW period as a typical example, and displayed them in Fig. S8. The formation of the coherent Kondo lattice can also be manifested from the evolution of the Kondo resonance close to the island edge. In a coherent nano-scale Kondo lattice, magnetic atoms at the inner and edge of the lattice have different quantum interference environments, resulting in a significant change in their Kondo resonances^49^. In our monolayer VSe_2_ island, the Kondo resonance inside the island is uniform, but becomes gradually suppressed upon approaching the island edge (Fig. S19d, g), conforming to the coherent Kondo lattice behavior.

Note that Kondo lattice hallmarks a hybridization gap, which however is undetected in many Kondo lattice systems^19,22^. This is because its tunneling spectrum is determined by two interfering tunneling paths from the STM tip between the itinerant electrons and the Kondo resonance (Supplementary Note 2). To manifest the hybridization gap, one needs to tune the tunneling ratio between the two interfering tunneling paths. Since the lattice constants of VSe_2_ (0.352 nm) and the NbSe_2_ substrate (0.345 nm) are slightly different, larger VSe_2_ islands could accumulate internal strain, whose local variations might potentially tune the tunneling ratio. Fig. S10a shows a typical large VSe_2_ island whose area size (462 nm^2^) is about twice larger than that of Fig. 1a (292 nm^2^). Indeed, tunneling spectra acquired along a line at interior of the VSe_2_ island indicate prominent variations (Fig. S10c). Some of the spectra exhibit a single Kondo peak (purple curve as a typical example, Fig. S10c, e), which is the same as that of Fig. 3. Importantly, most of the spectra clearly display a pair of split Kondo peaks [red curve as a typical example, Fig. S10c, e), which hallmark the hybridization gap, in nice agreement with the hybridization gap reported in previous studies^4,50^. These two types of low-energy excitation spectral shapes have been reproduced by the co-tunneling model with different tunneling ratios in Fig. S9b, c. Such hybridization gaps have been robustly observed in different VSe_2_ islands in different batch of samples, as shown in Fig. S11 for another two examples. Those observations firmly confirm the presence of the hybridization gap in our system, providing the clear-cut fingerprint of the coherent Kondo lattice. Strictly speaking, the co-tunneling model should be applied to fit the Kondo lattice spectrum and its temperature evolution. However, it contains many parameters and does not possess a simple analytical expression, making such fitting to the experimental spectrum less straightforward. Thus, in actual Kondo lattice material systems, the spectrum for single ion Kondo state is frequently used to fit the Kondo lattice spectrum for simplicity^17–20^.

To unveil the experimental observations, we performed the DFT calculations. Although a controversy exists about the intrinsic ferromagnetism in monolayer VSe_2_^51,52^, it is believed that CDW formation would cancel magnetism^53^. Hence, the existence of magnetic moment in the $\sqrt{3}\times \sqrt{3}$ VSe_2_ is unexpected. We have constructed a series of possible VSe_2_/NbSe_2_ heterostructures to capture the experimental features, with/without the inclusion of intercalated vanadium (V_i_). We have searched for many different stacking orders (Fig. S12) and found that the structure shown in Fig. 1d, e fits best to the experimental d*I*/d*V* maps, while other constructed structures show noticeable derivations (Fig. S12). The calculated local density of states (DOS) based on that structure also reasonably agrees with the experimental spectra (Fig. 2a). This gives an additional proof of the intercalated V atoms (Fig. S13). The intercalated V atoms profoundly influence the lattice and electronic structures of this system, as shown in Fig. S3. Although $\sqrt{3}\times \sqrt{3}$ CDWs are generally observed despite stacking order or intercalation, these CDW patterns are characterized by V anti-trimerization with C_3_ symmetry, resulting in two inequivalent V-V bonds ~3.7 Å and ~3.3 Å. The symmetry is same as monolayer, suggesting CDWs in vdW heterostructure are less influenced by interlayer interaction, as is also reflected in the electronic properties (Fig. S14b). However, the introduction of V_i_ under isosceles triangle V (formed by two bonds of 3.3 Å and one of 3.7 Å) in the vdW heterostructure would result in all V-V bonds unequal with obvious C_1_ symmetry, e.g., V_1_-V_2_ ~ 3.7 Å V_1_-V_3_ ~ 3.0 Å and V_2_-V_3_ ~ 3.2 Å (Fig. 1d). The electronic properties are also strongly influenced by V_i_ (Fig. S14c). Significant hybridizations can be found between V_i_ and VSe_2_/NbSe_2_ in the spin-up channel. Furthermore, amounts of charges transfer from V_i_ into both VSe_2_ and NbSe_2_ layers, which could be responsible for the reduction of the observed layer height.

This new CDW system possesses local magnetic moments inside the superlattice. As can be seen in the calculated spin density distribution (Fig. 1d). Within one unit cell of the CDW phase magnetic moment of V_i_ is as large as ~1${\mu }_{B}$ composed of mixing ${3d}_{{xy}}$ and ${3d}_{{z}^{2}}$ at the energy range −0.9 ~ −0.55 eV below *E*_F_ (Fig. S15), while the magnetic moment inside the VSe_2_ layer is ~ −0.9${\mu }_{B}$ mainly contributed by V_2_ (~ −0.7${\mu }_{B}$ composed of ${3d}_{{yz}}$ and ${3d}_{{z}^{2}}$ at the energy range −0.75 ~ −0.3 eV below *E*_F_ (Fig. S15)), opposite to V_i_. Importantly, the existence of V_i_ has also noticeably suppressed the magnetic exchange interaction between superlattice. Specifically, without V_i_ the calculated magnetic moments of the monolayer $\sqrt{3}\times \sqrt{3}$ VSe_2_ are ~ 3.3 ${\mu }_{B}$ (Fig. S14a), resulting in a relatively large ferromagnetic exchange interaction ~ 4 meV for nearest-neighboring unit cell. However, with V_i_ the magnetic moments decrease to ~ 0.9 ${\mu }_{B}$ partially caused by the electrons transferring from V_i_ to the unoccupied ${3d}_{{z}^{2}}$ orbitals (Fig. S14c) and the exchange interaction between superlattice is reduced to ~ 1 meV, destabilizing the long-range magnetic order and benefiting superconducting proximity effect. Overall, the magnetic moment array, an ingredient for Kondo lattice, could be contributed by both VSe_2_ and V_i_, but V_i_ is likely play a more important role as a result of flatter band dispersion (350 meV) and magnetic moments closer to ~ 1${\mu }_{B}$ than the V in VSe_2_ layer. Although the orbital bands forming the local moments are conventionally far from the Fermi level, they can be screened by the itinerant conduction electrons via spin-flip scattering, which involves a virtual hopping process as in the case of Mn_3_Sn^19^. Overall, the CDW phase triggered by the intercalated vanadium plays a vital role in the formation of Kondo lattice, indirectly influencing the interplay between the Kondo lattice and the proximity superconductivity.

**Fig. 4:**
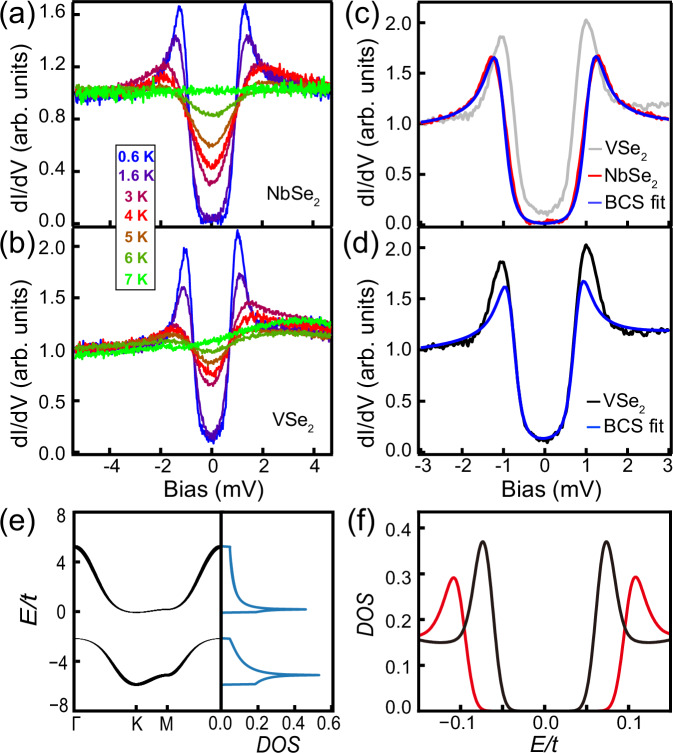
The proximity-induced SC gaps of √3 × √3 VSe_2_. **a**, **b** Temperature dependence of SC gaps measured on the NbSe_2_ substrate and the $\sqrt{3}\times \sqrt{3}$ VSe_2_, respectively. **c**, **d** SC spectra of NbSe_2_ and VSe_2_, and their respective corresponding BCS fittings (*V*_b_ = 5 mV, *I*_t_ = 200 pA, *V*_mod_ = 0.05 mV). **e** Band structure and corresponding DOS of the Kondo lattice model at $t=1,J=5.5$. The occupation number of the itinerant electron is ${n}_{c}=1.1$. The Kondo hybridization causes a heavy electron band near *E*_F_ characterized by a sharp resonance peak at $E=0.2$ in the DOS. The thickness of the band dispersion is proportional to the spectral weight of the conduction electrons. **f** DOS near *E*_F_ showing the SC gap of the Kondo lattice (black curve) to be compared with that of a simple one-band BCS superconductor (red curve) with gap ${\Delta }_{0}=0.108.$ The Kondo lattice shows a smaller superconducting gap $\Delta=0.074$, but an enhanced spectral weight of the coherence peaks. The small difference with experiment (${\Delta }_{{{\rm{Nb}}}{{{\rm{Se}}}}_{2}}=1.07$ meV, ${\Delta }_{{{\rm{V}}}{{{\rm{Se}}}}_{2}}=0.80$ meV) is due to the fact that the Kondo lattice model considered in our work is a simplified model with only one relevant itinerant electron band and only near-neighbor hopping between itinerant electrons is considered.

Having identified the Kondo lattice, we study its interaction with the superconductivity of the NbSe_2_ substrate. For single Kondo impurity, its exchange interaction with Cooper pairs induces Yu-Shiba-Rusinov (YSR) bound states inside the SC gap. While YSR states from single Kondo impurities have been extensively studied, the fate of Kondo lattice interacting with superconductivity remains unexplored in experiment. As such, we performed spectroscopic measurement to the SC gap at 0.6 K. As is seen in Fig. 4(a, b), the measured d*I*/d*V* spectra on the substrate and the VSe_2_ both show SC gaps. With increasing temperature, SC gap of the substrate becomes gradually suppressed and disappears completely at 7 K. Interestingly, while the SC gap size of VSe_2_ is smaller than that of the substrate, its gap follows a similar evolution trend as the substrate with an identical SC transition temperature, demonstrating the SC gap of VSe_2_ originates from the SC proximity effect of the substrate. The superconducting proximity decay length of our intercalated VSe_2_ film is estimated as 2.1 ~ 3.1 nm by an exponential fitting to the superconducting gap sizes of bare NbSe_2_ and monolayer VSe_2_ (Fig. S16), which is larger than the VSe_2_ films without intercalation^54^. In addition, the proximitized SC gap measured across multiple CDW periods is spatially uniform (Fig. S17). In the presence of an adsorbate whose species is currently unknown, the Kondo peak is locally suppressed, but the SC gap is kept unchanged, conforming to the proximity effect origin of the SC gap (Fig. S18) and substantiating the Kondo lattice formation, ruling out the presence of YSR states (Supplementary Note 3).

Finally, we evaluated the spectral shape of the proximitized SC gap. The SC gap of NbSe_2_ fully opens, whose spectral shape fits nicely to the BCS function, yielding a SC gap size of 1.07 meV (Fig. 4c). The measured gap size is slightly smaller than that of the pristine NbSe_2_, 1.20 meV, which is likely caused by the growth of VSe_2_. The gap of the monolayer VSe_2_, on the other hand, exhibits finite DOS within the gap and a pair of coherence peaks with higher intensity on the empty state (Fig. 4c, d). The asymmetric SC gap comes from the background introduced from the Kondo peak, which can be subtracted to restore the gap symmetry (Fig. S20). Notably, the SC gap of VSe_2_ exhibits obviously more enhanced coherence peaks than the conventional BCS function, implying prominent entanglement between superconductivity and the heavy electrons. To understand its unusual SC gap shape, we consider an effective Kondo lattice model in the $\sqrt{3}\times \sqrt{3}$ CDW phase of the monolayer VSe_2_, whose details are described in Methods. Here we summarize the key results in Fig.4e, f. In the normal state, the Kondo hybridization between local moments and itinerant electrons gives rise to a heavy electron band crossing *E*_F_, characterized by the sharp resonance peak in the density of state near *E*_F_. A hybridization gap in the density of states opens near *E*_F_. But the real system contains multiple conduction bands crossing *E*_F_, and therefore, in reality this gap is filled by other conduction bands that do not hybridize with local moments for symmetry reason. In our model, the SC gap ${\Delta }_{0}$ of the neighboring NbSe_2_ layer acts as an effective SC pairing potential and induces a SC gap $\Delta$<Δ_0_ in the VSe_2_ layer. The reduction of the SC gap is a direct consequence of strong competition between the superconductivity and Kondo hybridization, given both of them rely on formation of spin singlet pairs. The pairing state consists of electrons in the heavy electron band that participate in superconductivity when the temperature is below the SC transition temperature and make an additional contribution to the spectral weights. The hybridized nature of this band causes enhanced spectral weight of the coherence peak in the Kondo lattice model that deviates from the function of a simple one-band BCS superconductor.

## Discussion

In summary, we achieved growth of monolayer VSe_2_ on superconducting 2H-NbSe_2_ substrate. The monolayer VSe_2_ exhibits a new $\sqrt{3}\times \sqrt{3}$ CDW phase and more importantly forms a coherent Kondo lattice with an identical periodicity as the CDW pattern. Superconductivity is established in the Kondo lattice through proximity effect from the substrate. The proximitized SC gap exhibits more enhanced coherence peaks, whose spectral shape deviates from the function of one-band BCS superconductor, but is reproduced by model calculations with heavy electrons participating the pairing condensate. Our work realizes a new van der Waals heterojunction system exhibiting Kondo lattice behavior and coexisting proximitized superconductivity, which indicates that electrons from the coherent Kondo screening can take part in superconducting pairing. This could facilitate the understanding of the unconventional superconductivity in heavy fermion systems, wherein exchange interactions among electrons of Kondo screening are believed essential^55^.

## Methods

### Sample preparation

The bulk 2H-NbSe_2_ substrate was cleaved and further degassed at 450 °C and in an ultra-high vacuum chamber. After cleaving, the VSe_2_ films are grown by co-deposition of high-purity Se (purity 99.999%) and V (purity 99.95%) atoms from a Knudsen cell and an electron-beam evaporator, respectively, while the 2H-NbSe_2_ substrate is kept at a suitable temperature for 15 minutes. Different substrate temperatures (310°C ~ 435°C) correspond to different V-Se compounds. The temperature required for the $\sqrt{3}\times \sqrt{3}$ VSe_2_, which is the focus of this study, is slightly higher than 400 °C.

### STM measurement

The experiments are performed with a custom-made Unisoku STM (1300). An electrochemically etched W wire is used as the STM tip. Prior to measurements, the tip is characterized on a Ag (111) multilayer film grown on a Si (111) substrate, which has been cleaned by several cycles of flashing to 1500 K. The tunneling spectra are measured using a standard lock-in detection technique with a modulation voltage at 983 Hz.

### DFT calculation

Our first-principles calculations are performed with Vienna ab initio simulation package (VASP)^35^ where projector augmented wave method is adopted^56^. The exchange-correlation term is treated with the Perdew-Burke-Ernzerhof (PBE) form of the generalized gradient approximation^57^. The 3*p*^6^3*d*^4^4*s*^1^, 4*p*^6^4*d*^4^5*s*^1^ and the 4*s*^2^4*p*^4^ electrons are treated as valence electrons for V, Nb and Se, respectively. We construct a two-layer VSe_2_/NbSe_2_ heterojunction in our DFT calculation, which can capture the major characters of experimental monolayer-VSe_2_/bulk-NbSe_2_ structure. A vacuum layer of about 15 Å is added to avoid interactions between periodical layers and DFT-D3 method of Grimme with zero-damping function^58^ is adopted to correct the van der Waals force. Gaussian smearing of 0.05 eV is used in our calculations. Energy cutoff of 500 eV and *k* mesh of $8\times 8\times 1$ in Brillouin zone (BZ) are adopt for structural relaxation of the heterojunction, with force on each atom is less than 0.005 eV/Å and the convergence criterion of total energy being 10^-6 ^eV. Increased *k* mesh of $12\times 12\times 1$ in BZ and convergence criterion of total energy 10^-7 ^eV are adopted in static calculations. We determine the intercalation structure by investigating the simulated d*I*/d*V* maps with experiments. The simulation of d*I*/d*V* maps is based on the summation of DOS at corresponding energy *E*_b_ within range ${E}_{b}\pm 0.01$ eV and a distance of about 3 Å from the surface. The effect of spin orbital coupling (SOC) is negligible in our system and not considered.

### Theoretical model

The interplay of Kondo hybridization and proximitized superconductivity has been investigated by a Kondo lattice model within a self-consistent mean-field approach.

In principle, the CDW order leads to a multi-band electronic structure. A band close to half-filling may experience stronger electron correlations and becomes localized, via an orbital-selective mechanism. Electrons in this localized band can then be approximated as local magnetic moments because charge excitations are heavily suppressed and gapped. On the other hand, electrons in other bands are still itinerant. To understand the low-energy physics of this system consisting of both itinerant and localized electrons, here we consider an effective Kondo lattice model defined on the $\sqrt{3}\times \sqrt{3}$ triangular superlattice, which corresponds to the lattice structure of the $\sqrt{3}\times \sqrt{3}$ CDW phase of the monolayer VSe_2_. The Hamiltonian of the model reads as

$$H=t{\sum}_{\left\langle i,j\right\rangle,\sigma }{c}_{i{\sigma }}^{{\dagger} }{c}_{j\sigma }+\frac{J}{2}{\sum}_{i,\sigma,{\sigma }^{{\prime} }}{{{\boldsymbol{S}}}}_{i}\cdot {c}_{i\sigma }^{{\dagger} }{{{\boldsymbol{\tau }}}}_{\sigma {\sigma }^{{\prime} }}{c}_{i\sigma }-{\Delta }_{0}{\sum}_{k}\left({c}_{k\uparrow }^{{\dagger} }{c}_{-k\downarrow }^{{\dagger} }+h.c.\right)$$

where ${c}_{i\sigma }^{{\dagger} }$ creates an electron of spin $\sigma$ at the superlattice site *i* in the itinerant band, ${{{\boldsymbol{S}}}}_{i}$ denotes the $S=1/2$ local magnetic moment induced in the CDW phase, ${{\boldsymbol{\tau }}}$ is the Pauli matrix, $t$ refers to the nearest neighbor hopping between itinerant electrons, and $J$ is the Kondo coupling. Note that our purpose is to capture the essential physics of the Kondo lattice. Therefore, we consider a single conduction band relevant to the Kondo hybridization, which can be approximately described by a tight-binding model with the nearest neighbor hopping. The realistic electronic structure of the monolayer VSe_2_ may be more complicated, which can be described by including other conduction bands and with further neighboring terms. We assume the SC gap in the NbSe_2_ layers is ${\Delta }_{0}$. This conventional BCS type gap acts as an attractive SC pairing potential to itinerant electrons in the monolayer VSe_2_ via the proximity effect.

To understand the interplay of Kondo hybridization and superconductivity in this model, we first rewrite the local spin operator in the pseudo-fermion representation: ${{{\boldsymbol{S}}}}_{i}=\frac{1}{2}{f}_{i\sigma }^{{\dagger} }{{{\boldsymbol{\tau }}}}_{\sigma {\sigma }^{{\prime} }}{f}_{i{\sigma }}$, where ${f}_{i\sigma }$ satisfies the fermionic commutation. For an exact representation of the $S=1/2$ local moment, we employ a constraint ${\sum }_{\sigma }{f}_{i\sigma }^{{\dagger} }{f}_{i\sigma }=1$ via introducing a Lagrange multiplier ${\epsilon }_{f}$ in the Hamiltonian. In the pseudo-fermion representation, the local moment corresponds to a flat pseudo-fermion band at the Fermi energy. To handle the Kondo coupling term, we then adopt a mean-field decomposition

$${\sum}_{i,\sigma,{\sigma }^{{\prime} }}{{{\boldsymbol{S}}}}_{i}\cdot {c}_{i\sigma }^{{\dagger} }{{{\boldsymbol{\tau }}}}_{\sigma {\sigma }^{{\prime} }}{c}_{i\sigma }\approx V{\sum}_{i,\sigma }{c}_{i,\sigma }^{{\dagger} }{f}_{i\sigma }$$

where $V=\langle {f}_{i,\sigma }^{{\dagger} }{c}_{i\sigma }\rangle$, parametrizes the Kondo hybridization. For simplicity, we assume translational symmetry of $V$. In our calculation, $V$ and ${\epsilon }_{f}$ are determined in a self-consistent way. Given the bilinear coupling between *c* and *f* fermions, it is expected that the SC pairing between itinerant electrons can induce pairing within the pseudo-fermions. We therefore introduce an additional mean field ${\Delta }_{f}=\langle {f}_{k\uparrow }{f}_{-k\downarrow }\rangle$, which is self-consistently determined together with $V$ and ${\epsilon }_{f}$.

In the normal state, we can set ${\Delta }_{0}=0$. Our results of the band structure and the corresponding density of states projected to the conduction electrons at $t=1,J=5.5$ are shown in Fig. 4e of the main text. It is clear that the Kondo coupling causes a finite hybridization between the itinerant and pseudo fermions. As a consequence of the Kondo hybridization, the flat pseudo-fermion band becomes weakly dispersive, forming a heavy electron band. This heavy electron band is characterized by a sharp resonance peak of the density of states near the Fermi energy. This Kondo resonance peak is also resolved in experiment. A rough estimate from the CDW gap gives *t* ~ 50 meV. With this, our calculation suggests that the heavy band should be very close to the Fermi level at ~10 meV. The energy of the heavy band is in reasonable agreement with the experimentally observed Kondo resonance at around 5 meV. Note that in our model a finite hybridization gap opens below the Fermi energy. In reality, such a hybridization gap is likely not observed because the gap could be filled by other conduction bands across the Fermi energy that does not hybridize with local moments for symmetry reasons.

When ${\Delta }_{0}$ is turned on, the model can be solved via a Bogoliubov transformation. As discussed above, in the presence of Kondo hybridization this is an effective two-band system, and the excitation energies in the SC state are ${E}_{k}^{\pm }$. The induced SC gap $\Delta$ can be calculated from the lowest quasiparticle excitation energy ${E}_{k}^{-}$ at the Fermi energy, e.g., $\Delta={E}_{k={k}_{F}}^{-}$. The density of states can be obtained from $\rho={\sum }_{k,\alpha=\pm }[\delta (\omega -{E}_{k}^{\alpha })+\delta (\omega+{E}_{k}^{\alpha })]$.

The calculated density of states near the Fermi energy (set to zero) in the Kondo lattice at ${\Delta }_{0}=0.1,t=1,J=5.5$ is plotted in Fig. 4f of the main text. Compared to that of a one-band superconductor with SC gap ${\Delta }_{0}$, we find two major differences. First, the induced SC gap $\Delta$ in the Kondo lattice is smaller than ${\Delta }_{0}$. The reduction of the SC gap is understood as follows: An itinerant electron may either participate in superconductivity by forming a spin singlet Cooper pair with another itinerant electron, or screen the local moment by forming a Kondo singlet. These two processes are in direct competition. As a result, the SC gap is reduced in the presence of Kondo hybridization. The other difference is that the spectral weight of the coherence peak is enhanced in the Kondo lattice model compared to that in a simple BCS superconductor. Note that the band near the Fermi energy is the heavy electron band. This heavy electron band contains a significant portion of *f*-fermions. The participation of these electrons to superconductivity naturally causes enhancement of spectral weight. Moreover, as mentioned above, the Kondo lattice can be viewed as a two-band system consisting of *c*- and *f*-fermions. Therefore, the gap function is more complicated than and can deviate from that of a simple one-band superconductor.

## Supplementary information

Supplementary Information

Peer Review file

## Data Availability

The data that support the findings of this study are available from the corresponding author upon request.

## Code availability

The code that supports the findings of this study is available from the corresponding author upon request.
