# Supplementary material for: Artificial superconducting Kondo lattice in a van der Waals heterostructure
Source: Nat Commun. 2024 Oct 11;15:8797. doi: 10.1038/s41467-024-53166-9 (PMC11470022; doi:10.1038/s41467-024-53166-9)
Supplement: Supplementary file 1 — Supplementary Information [file 41467_2024_53166_MOESM1_ESM.pdf]

Supplemental Materials for

Artificial superconducting Kondo lattice in a van der Waals heterostructure

Kai Fan[1#], Heng Jin[2,3#], Bing Huang[2,3#], Guijing Duan[4], Rong Yu[4], Zhen-Yu Liu[1], Hui-Nan Xia[1], Li-Si Liu[1], Yao Zhang[1], Tao Xie[1], Qiao-Yin Tang[1], Gang Chen[1], Wen-Hao Zhang[1], F. C. Chen[5], X. Luo[5], W. J. Lu[5], Y. P. Sun[6,5,7], Ying-Shuang Fu[1,8†]

[1]School of Physics and Wuhan National High Magnetic Field Center, Huazhong University of Science and Technology, Wuhan 430074, China

[2]Department of Physics, Beijing Normal University, Beijing 100875, China

[3]Beijing Computational Science Research Center, Beijing 100093, China

[4]Department of Physics and Beijing Key Laboratory of Opto-electronic Functional Materials and Micro-nano Devices, Renmin University of China, Beijing 100872, China

[5]Key Laboratory of Materials Physics, Institute of Solid State Physics, Chinese Academy of Sciences, Hefei, 230031, China

[6]High Magnetic Field Laboratory, Chinese Academy of Sciences, Hefei, 230031, China

[7]Collaborative Innovation Center of Advanced Microstructures, Nanjing University, Nanjing, 210093, China

[8]Wuhan Institute of Quantum Technology, Wuhan 430206, China

#These authors contribute equally to this work.

[†]Email: yfu@hust.edu.cn

Contents

Note 1: Evidence and role of intercalated V atoms

Note 2: Influence of tunneling path ratios on tunneling spectra

Note 3: Exclusion of YSR states

Fig. S1: Sample growth.

Fig. S2: STM topography of the as-grown monolayer VSe2 with different growth parameters.

Fig. S3: Comparison of monolayer VSe_2 with and without V atom intercalation.

Fig. S4: Spatially-dependent large range d*I*/d*V* spectra of $\sqrt{3} \times \sqrt{3}$ VSe_2.

Fig. S5: Anti-phase relation for CDW gap edges.

Fig. S6: Constant-current STM images of $\sqrt{3} \times \sqrt{3}$ VSe_2 at different bias voltages.

Fig. S7: Constant-height d*I*/d*V* mappings of $\sqrt{3} \times \sqrt{3}$ VSe_2 at different bias voltages.

Fig. S8: Normalized d*I*/d*V* in a CDW period.

Fig. S9: Influence of tunneling path ratios on tunneling spectra.

Fig. S10: Hybridization gap and superconducting proximity gap spectra in large VSe_2 island.

Fig. S11: Hybridization gap spectra on another two VSe_2 islands.

Fig. S12: Simulated d*I*/d*V* maps of different stacking structure with/without interstitial vanadium at typical bias voltage.

Fig. S13: Comparison between experimental d*I*/d*V* maps and DFT-simulated maps.

Fig. S14: Comparation of the electronic properties of between $\sqrt{3} \times \sqrt{3}$ monolayer VSe_2, $VSe_2/NbSe_2$ vdW two-layer and the V-intercalated $VSe_2/NbSe_2$ structure.

Fig. S15: 3*d* orbital-projected band structure of V_i and V in the intercalated structure.

Fig. S16: Estimation of the decay lengths of superconducting proximity effect.

Fig. S17: Spatial distribution of SC gap at homogeneous regions.

Fig. S18: Influence of an adsorbate on Kondo resonance peak and SC gap.

Fig. S19: Comparison of spectra at the inner and edge of VSe_2 island.

Fig. S20: Removing the tilted background of the SC gap of $\sqrt{3} \times \sqrt{3}$ VSe_2.

Fig. S21: Different shapes of Kondo lattice spectra and their superconducting proximity gaps.

Fig. S22: Excluding quantum size effects in VSe_2 islands.

Note 1: Evidence and role of intercalated V atoms

To confirm the existence of V interstitial atoms, cross-sectional TEM imaging of the $VSe_2/NbSe_2$ heterostructure is the most direct method. However, the coverage of our $\sqrt{3} \times \sqrt{3}$ VSe_2 islands, that is estimated as 5%, is too small to perform such measurement. Nevertheless, the existence of V intercalated atoms can be evidenced from the following two aspects. First, STM topography at characteristic energies of the doped impurity level could give experimental proof of intercalated V atoms. In our case, the intercalated V atoms hybridize with the heterostructure, making their defect level mixed into the local density of states (LDOS) of the sample. Despite of the hybridization, the intercalated V atoms contribute to the LDOS that are visualized from spectroscopic mappings at corresponding energies. As shown in Figs. 2 and S13(c), spectroscopic mappings of the $\sqrt{3} \times \sqrt{3}$ VSe_2 indicates the Se trimer unit breaks its three-fold symmetry. This indicates the existence of intercalated V atoms beneath one of the Se trimer atoms, as is schematically shown in Fig. 1e. The spectroscopic mappings are further substantiated from our DFT-simulated in Figs. S13(a, b), giving additional proof of the intercalated V atoms. Second, intercalation of V

atoms can also be deduced from the perspective of growth kinetics by comparing sample morphology under different growth conditions. As shown in Figs. S2 (a, d), under growth condition of Se-rich and low substrate temperature, the VSe_2 monolayers are grown into a well-known CDW pattern of coexisting $2 \times \sqrt{3}$ and $\sqrt{7} \times \sqrt{3}$ periodicity, conforming to previous study of monolayer VSe_2 on graphene substrate [1-3]. With the reduction of Se flux and increase of substrate temperature, the growth conditions are promoted into a V-rich environment, small regions of $\sqrt{3} \times \sqrt{3}$ pattern emerge as separated patches [Figs. S2(b, e)]. Further reduction of Se flux and increasing substrate temperature results in the formation of uniform $\sqrt{3} \times \sqrt{3}$ pattern throughout the entire VSe_2 monolayer films [Figs. S2(c, f)]. Note that elevating the substrate temperature in effect increases the desorption of Se atoms on the surface and meanwhile increases the thermal energy of V atoms, facilitating the intercalation of V atoms into the van der Waals gap of the heterojunction interface.

To investigate the energetic stability of the intercalated structure we've also calculated the formation energy of the intercalated structure with the following expression $E_{form} = E_{int} - (E_{vdW} + E_V)$, in which E_{int}, E_{vdW} and E_V are the energy of the intercalated structure, vdW two-layer structure and solid elemental vanadium, respectively. The DFT-calculated E_{form} is ~ -1.64 eV/V_i. The negative value of E_{form} indicates a tendency to form the intercalated structure under the condition that sufficient vanadium is supplemented during the growth process, which agrees with our experiments.

To evaluate the role of intercalated V atoms, we compare the topography, CDW pattern, and spectra on monolayer VSe_2 with and without V atom intercalation, as shown in Fig. S3. The non-intercalated VSe_2 displays a coexisting CDW pattern of $2 \times \sqrt{3}$ and $\sqrt{7} \times \sqrt{3}$

[Fig. S3(a)], similar to that grown on HOPG substrate reported in Ref. 37. The intercalated VSe_2, on the other hand, exhibits a CDW pattern of $\sqrt{3} \times \sqrt{3}$ [Fig. S3(d)], which is distinct to that of the non-intercalated case. The spectra of the non-intercalated and intercalated VSe_2 are also different. As shown in Fig. S3(c), the non-intercalated VSe_2 exhibit a dip feature at the Fermi level, which is similar to that measured on VSe_2 grown on HOPG substrate reported previously [1]. In contrast, the intercalated VSe_2 exhibits a CDW gap ranging from -130 mV to 25 mV. More importantly, the intercalated VSe_2 has a pronounced Kondo peak emerging around the Fermi level, which is absent in the non-intercalated case. The presence of the intercalated V atoms enhances the coupling between monolayer VSe_2 and the $NbSe_2$ substrate, which leads to a reduction in their interlayer spacing. Indeed, as seen in Figs. S3(b, e), the apparent height of monolayer VSe_2 is measured as 0.62 nm and 0.58 nm for the non-intercalated and intercalated case, respectively. It is worth to mention that such intercalation induced reduction in interlayer spacing is also reproduced by our DFT calculations, as shown in Figs. S3(g-i). The calculated height of $2 \times \sqrt{3}$ and $\sqrt{7} \times \sqrt{3}$ vdW structures are 0.61 and 0.62 nm while the intercalated $\sqrt{3} \times \sqrt{3}$ structure has a height of 0.58 nm, perfectly agreeing with the experimental measurement.

Note 2: Influence of tunneling path ratio on tunneling spectra

The Kondo-lattice hallmarks a hybridization gap, which should be ideally detected. However, STS measurement doesn't possess momentum resolution to directly detect the hybridization gap. More importantly, for STS measurements, the tunneling spectrum of a Kondo lattice should be manifested as a Fano line shape "because of the presence of two interfering tunneling paths from the STM tip, one directly into the itinerant electrons, and the other indirectly through the Kondo resonance" [4].

In order to clearly represent the above effect, the cotunneling model proposed for the Kondo lattice system is used to depict the evolution of the spectral shape with the two tunneling path ratios. As described in Refs. 5 and 6, the co-tunneling model considers an electron-like conduction band $\mathrm{E}_k^c = 2\mathrm{t}(\cos \mathrm{k}_x + \cos \mathrm{k}_y) - \mu$ [dashed blue in Fig. S9(a)] and a heavy flat band $\mathrm{E}_k^f = -2\chi_0(\cos\mathrm{k}_x + \cos\mathrm{k}_y) + \varepsilon_0^f$ [dashed pink in Fig. S9(a))]near the Fermi surface, where t is nearest neighbor hopping of the conduction electrons, χ_0 is nearest site spin correlation, μ is chemical potential and ε_0^f is the position of the heavy band respect to Fermi energy. Two heavy fermionic bands $E_k^{\pm} = \frac{E_k^c+E_k^f}{2} \pm \sqrt{\left(\frac{E_k^c-E_k^f}{2}\right)^2 + \nu^2}$are obtained when coherent Kondo shielding is considered, where ν depicts the hybridization amplitude between the light and flat bands. As a result, the differential conductance can be described by $dI/dV \propto \sum_{i,j=1}^{2}[tImG(k,\omega)]_{ij}$, where t represents the tunneling ratio between the conduction band and the heavy flat band, and the full Green's function describing the hybridization between the above two bands. According to this model, different spectral shapes can be obtained by using different tunneling amplitudes for the heavy and light bands ($t_f/t_c = 5, 0.17, 0.002$), as shown in Figs. S9(b-d). It is clearly seen that as the heavy band tunneling amplitude t_f increases [such as $t_f/t_c = 0.17$, Fig. S9(c)], differential conductance exhibits the shape of the Kondo resonance peak without showing a significant hybridization energy gap. In contrast, when the conducting band tunneling amplitude t_c increases [such as $t_f/t_c = 0.002$, Fig. S9(d)], the differential conductance exhibits more features of the hybridization energy gap. Note that when the heavy-band tunneling amplitude is particularly large, the differential conductance shows a pair of split peaks [such as $t_f/t_c = 5$, Fig. S9(b)].

As such, the hybridization gap, that is most prominent in the itinerant electron band, cannot be observed in the case of dominate tunneling through the Kondo resonance. This is exactly our case showing a Kondo resonance peak, since the magnetic impurity lattice is closer to the STM tip, while the itinerant electrons in the $NbSe_2$ substrate are farther to the tip. As are also exemplified in Ref. 7 and 8, only Kondo resonance peaks were observed in many Kondo lattice systems, instead of the expected hybridization gap.

Note 3: Exclusion of YSR states

In our study, the Kondo temperature T_K is much higher than the superconducting transition temperature T_C. For a single magnetic impurity, the YSR state stabilizes very close to the superconducting gap edge in the limit of $T_K \gg T_C$. However, we can rule out that the enhanced SC coherence peaks is due to the presence of the YSR states for several reasons:

(1) As we already mentioned above that the spatial distribution of the Kondo resonance is uniform throughout the entire VSe_2 film. This would suggest a very high impurity concentration (one per $\sqrt{3} \times \sqrt{3}$ unit cell) of the Kondo impurities. If the change of the superconducting gap were caused by the presence of YSR states, for such a high impurity concentration, the superconducting gap would already be fully filled by the impurity band (see discussions of Ref. 9), but not just slightly narrowed.

(2) For YSR states, spectral weight of the particle- and hole-like excitation is influenced by the Coulomb potential, which conventionally breaks particle-hole symmetry [10]. However, this is in direct contrast to our experimental result, which exhibits almost symmetric spectrum in the superconducting state with only a slightly higher coherent peak at positive bias. the slightly asymmetric superconducting gap comes from the background

introduced from the Kondo peak, which can be subtracted to restore the gap symmetry, as is seen from Fig. S20. In addition, the two enhanced peaks measured across multiple CDW periods is spatially uniform (Fig. S17). Those observations are incompatible with the YSR origin of the two enhanced peaks, but suggest they are coherence peaks of proximity superconductivity.

(3) The presence of YSR states is also ruled out from the Kondo state and the superconductivity response to adsorbates and island boundaries. As shown in Fig. S18, the Kondo peak is locally suppressed at an adsorbate site of monolayer VSe_2, but the two enhanced peaks remain unchanged. This observation is in conflict with the scenario of non-interacting single ion Kondo state, because the YSR state at the adsorbate site would be distinctly different to the surrounding regions. Similarly, the Kondo resonance near the island boundary is significantly altered, but the measured superconductivity is consistent with the island inner [Figs. S19 (e, h)]. On the contrary, the interplay of Kondo lattice with superconductivity induces a superconducting proximity gap to monolayer VSe_2, whose coherence length is order of magnitude larger than the adsorbate, well explaining the uniform superconducting gap across the adsorbate.

(4) We further show three spectra acquired near a step edge in Fig. S21, showing strong variation of Kondo spectra. Specifically, the spectrum in pink has no Kondo peak, whose superconducting gap is completely symmetric, but still cannot fit into the BCS form. The spectrum in green has two split Kondo peaks with the right peak locating exactly at the Fermi level, whose superconducting gap is also symmetric. The spectrum in black shows slight asymmetric superconducting gap because its Kondo peak is above the Fermi level. Such observations further demonstrate the superconducting gap asymmetry is simply an

influence from the background, instead of the Shiba band. We would like to stress again that the superconducting gap in pink, despite of its absent Kondo peak, is identical to that in green. This clearly excludes the possibility of Shiba band, which sensitively depends on the Kondo feature.

(5) These points show that our results cannot be explained by effects of isolated Kondo impurities. On the other hand, the experimental finding that both coherent peaks are largely enhanced than those of the pristine $NbSe_2$ actually provides evidence for the coherent Kondo lattice behavior. The enhanced spectral weights of the coherent peaks indicate that the electrons on the intercalated V sites are delocalized and participate in superconductivity. Such an effect is only possible on a Kondo lattice where the local moments exhibit coherent behavior via hybridizing to itinerant electrons. As for the relevant temperature regime, the coherent Kondo temperature, T_{coh} is conventionally orders of magnitude larger than T_C in many heavy fermion superconductors [11]. The fact that $T_{coh} >> T_C$ leaves a broad temperature window for the formation of coherent Kondo lattice already above T_C.

Supplementary references:

[1] P. K. J. Wong, W. Zhang, F. Bussolotti, X. M. Yin, T. S. Herng, L. Zhang, Y. L. Huang, G. Vinai, S. Krishnamurthi, D. W. Bukhvalov, Y. J. Zheng, R. Chua, A. T. N'Diaye, S. A. Morton, C. Y. Yang, K. H. O. Yang, P. Torelli, W. Chen, K. E. J. Goh, J. Ding, M. T. Lin, G. Brocks, M. P. de Jong, A. H. C. Neto, and A. T. S. Wee. Evidence of Spin Frustration in a Vanadium Diselenide Monolayer Magnet. *Adv. Mater.* **31**, 1901185(2019).

[2] J. G. Feng, D. Biswas, A. Rajan, M. D. Watson, F. Mazzola, O. J. Clark, K. Underwood, I. Markovic, M. McLaren, A. Hunter, D. M. Burn, L. B. Duffy, S. Barua, G.

Balakrishnan, F. Bertran, P. Le Fevre, T. K. Kim, G. van der Laan, T. Hesjedal, P. Wahl, and P. D. C. King. Electronic Structure and Enhanced Charge-Density Wave Order of Monolayer VSe_2. *Nano Lett.* **18**,4493−4499(2018).

[3] G. Duvjir, B. K. Choi, I. Jang, S. Ulstrup, S. Kang, T. T. Ly, S. Kim, Y. H. Choi, C. Jozwiak, A. Bostwick, E. Rotenberg, J. G. Park, R. Sankar, K. S. Kim, J. Kim, and Y. J. Chang. Emergence of a Metal−Insulator Transition and High-Temperature Charge-Density Waves in VSe_2 at the Monolayer Limit. *Nano Lett.* **18**, 5432−5438(2018).

[4] P. Aynajian, E. H. da Silva Neto, C. V. Parker, Y. K. Huang, A. Pasupathy, J. Mydosh and A. Yazdani. Visualizing the formation of the Kondo lattice and the hidden order in URu_2Si_2. *PNAS* 107, 10383 (2010).

[5] Z. Y. Liu, H. Jin, Y. Zhang, K. Fan, T. F. Guo, H. J. Qin, L. F. Zhu, L. Z. Yang, W. H. Zhang, B. Huang and Y. S. Fu. Charge-density wave mediated quasi-one-dimensional Kondo lattice in stripe-phase monolayer 1T-$NbSe_2$. *Nat Commun* **15**, 1039 (2024).

[6] P. Aynajian, E. H. da Silva Neto, A. Gyenis, R.E. Baumbach, J.D. Thompson, Z. Fisk, E.D. Bauer, and A. Yazdani. Visualizing heavy fermions emerging in a quantum critical Kondo lattice. *Nature* **486**, 201–206(2012).

[7] S.S. Zhang, J. Yin, M. Ikhlas, H. Tien, R. Wang, N. Shumiya, G. Chang, S.S. Tsirkin, Y. Shi, C. Yi, Z. Guguchia, H. Li, W. Wang, T. Chang, Z. Wang, Y. Yang, T. Neupert, S. Nakatsuji, and M.Z. Hasan. Many-Body Resonance in a Correlated Topological Kagome Antiferromagnet. *Phys. Rev. Lett.* **125**, 046401(2020).

[8] S. Shen, C. Wen, P. Kong, J. Gao, J. Si, X. Luo, W. Lu, Y. Sun, G. Chen, S. Yan. Inducing and tuning Kondo screening in a narrow-electronic-band system. *Nat. Commun.* **13**, 2156 (2022).

[9] A. V. Balasky, I. Vekhter and J. X. Zhu. Impurity-induced states in conventional and unconventional superconductors. *Rev. Mod. Phys.* 78, 373 (2006)

[10] B. W. Heinrich, J. I. Pascual and K. J. Franke.Prog. Single magnetic adsorbates on s-wave superconductors. *Prog. Surf. Sci.* 93, 1-19 (2018)

[11] F. Steglich and S. Wirth. Foundations of heavy-fermion superconductivity: lattice Kondo effect and Mott physics. *Rep. Prog. Phys.* 79, 084502 (2016)

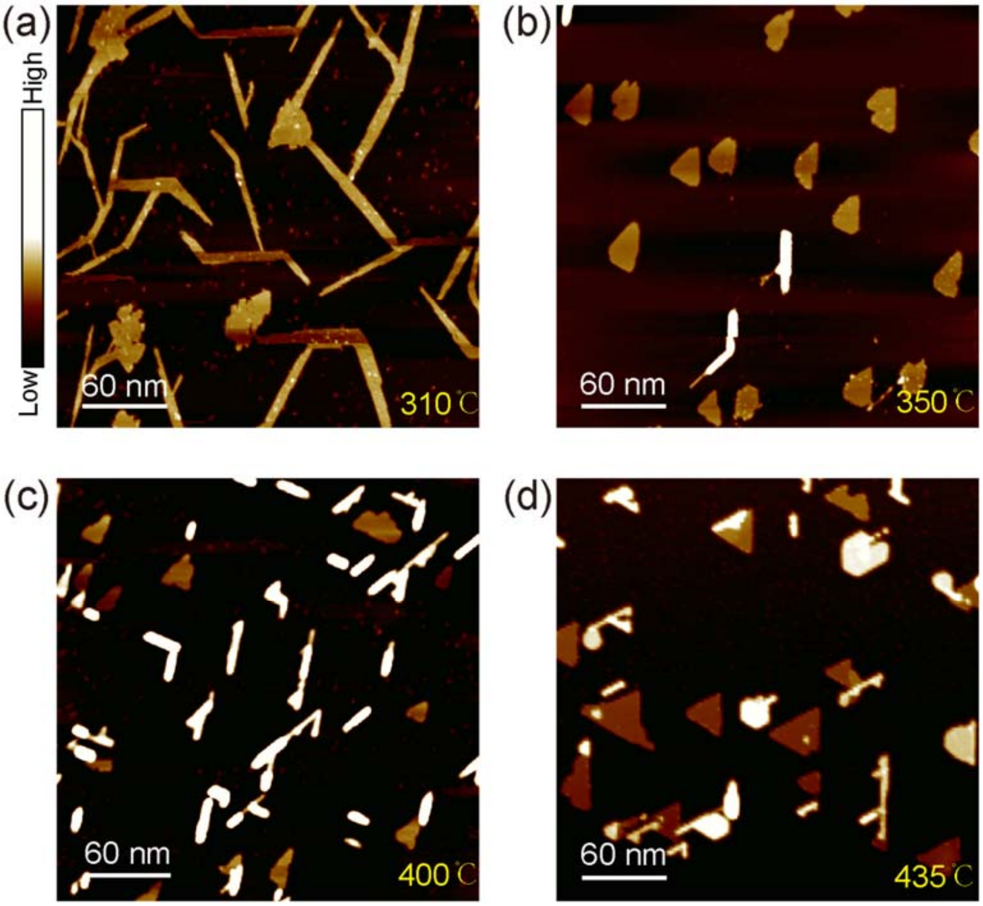

Fig. S1 Sample growth. (a-d) STM topography of the as grown sample with different growth parameters**.** Imaging conditions: (a) V_b = 1.0 V, I_t = 20 pA; (b,c) V_b = 1.0 V, I_t = 10 pA; (d) V_b = 1.0 V, I_t = 5 pA

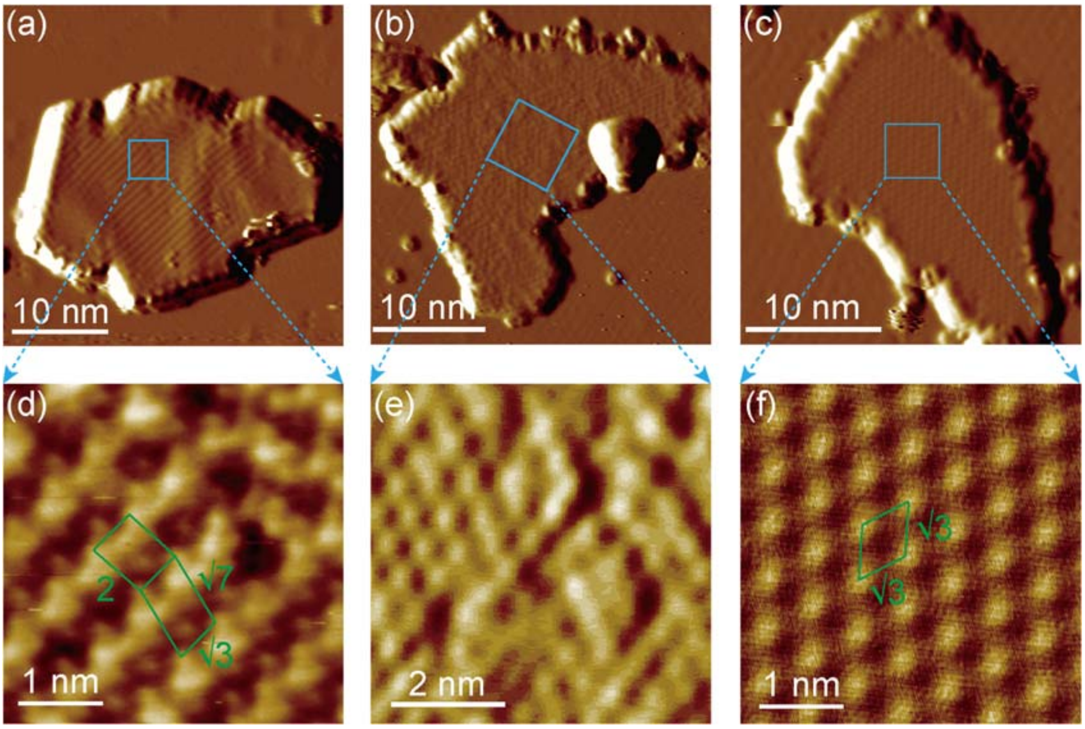

Fig. S2 STM topography of the as-grown monolayer VSe_2 with different growth parameters. (a) STM topography (V_b = 1 V, I_t = 10 pA) of a monolayer VSe_2 island with coexisting $2 \times \sqrt{3}$ and $\sqrt{7} \times \sqrt{3}$ CDW phases. (b) STM topography (V_b = 1.2 V, I_t = 10 pA) of a monolayer VSe_2 island showing separated small patches of $\sqrt{3} \times \sqrt{3}$ areas. (c) STM topography (V_b = -1 V, I_t = 10 pA) of a monolayer VSe_2 island with a complete and uniform $\sqrt{3} \times \sqrt{3}$ phase. The growth conditions of VSe_2 are 350°C for (a), 400°C for (b), 435°C for (c). (d-f) Magnified STM images of the area marked by the respective blue rectangles in (a-c).

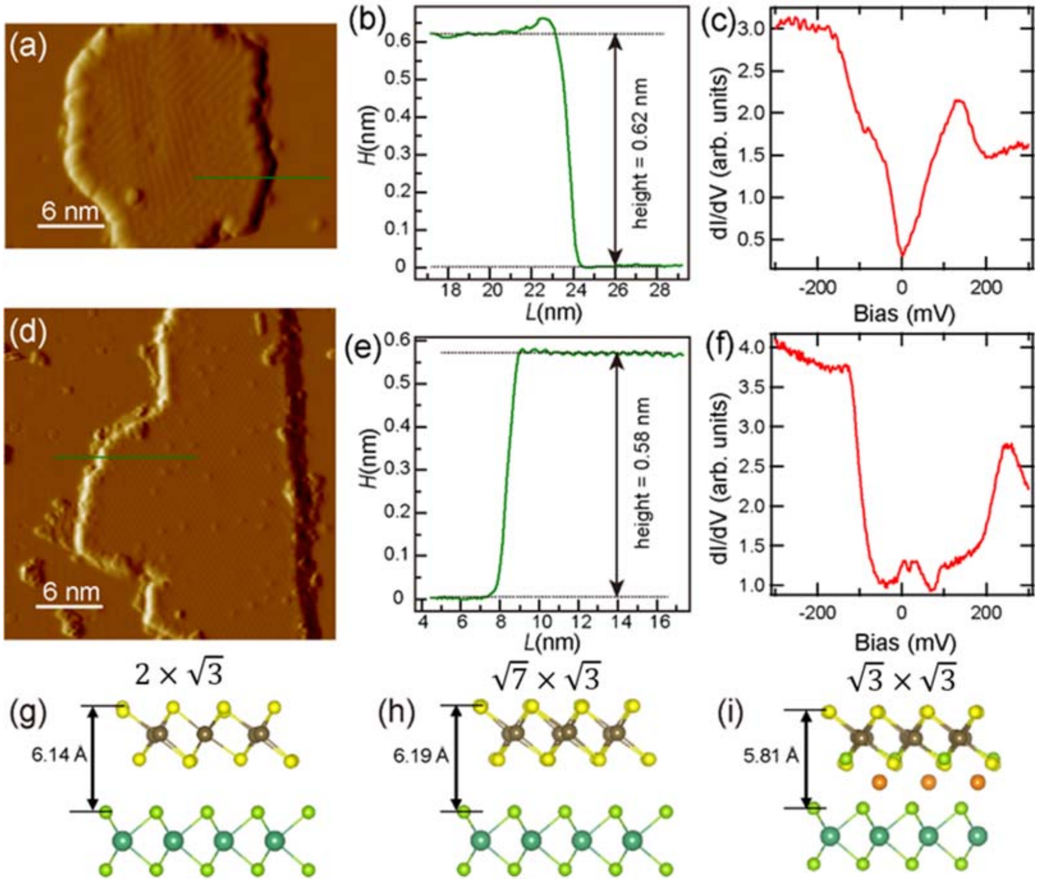

Fig. S3 Comparison of monolayer VSe_2 with and without V atom intercalation. (a, d) STM image of monolayer VSe_2 on $NbSe_2$ substrate with coexisting $2 \times \sqrt{3}$ and $\sqrt{7} \times \sqrt{3}$ phase (a), and $\sqrt{3} \times \sqrt{3}$ phase (d). (b) [(e)] Line profile showing apparent height of monolayer VSe_2 obtained along the green line in (a) [(d)]. (c, f) Typical dI/dV spectra for the coexisting $2 \times \sqrt{3}$ and $\sqrt{7} \times \sqrt{3}$ phase (c), and $\sqrt{3} \times \sqrt{3}$ phase (f), respectively. (g-i) DFT-calculated monolayer height of VSe_2 with (g) $2 \times \sqrt{3}$, (h) $\sqrt{7} \times \sqrt{3}$ and (i) intercalated $\sqrt{3} \times \sqrt{3}$ CDW patterns.

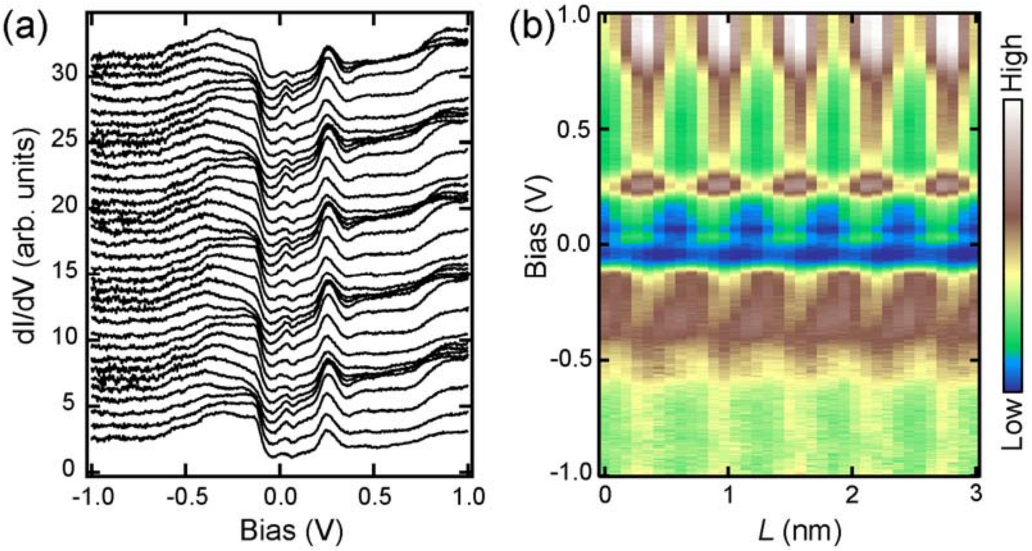

Fig. S4 Spatially-dependent large range d*I*/d*V* spectra of $\sqrt{3}\times\sqrt{3}$ VSe_2. (a,b)2D conductance plots and corresponding d*I*/d*V* spectra for large energy range measured along the white line in Fig. 3(d). The spectra are vertically offset for clarity. Spectral conditions: V_b = -1.0V, I_t = 200 pA, V_{mod} = 20 mV.

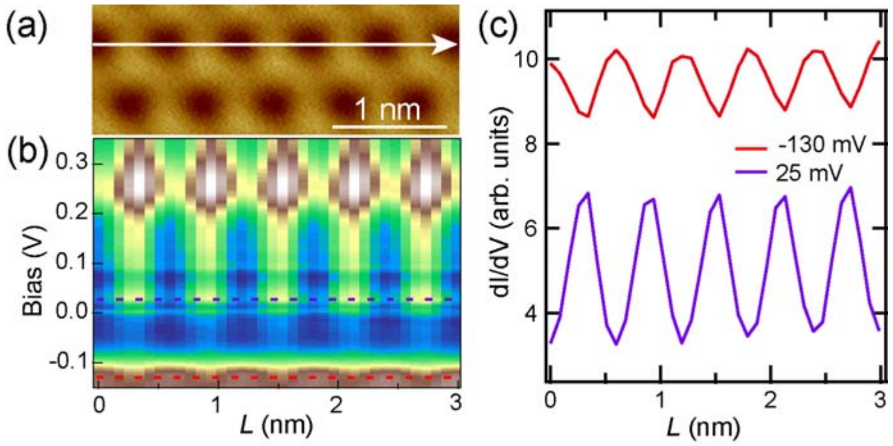

Fig. S5. Anti-phase relation for CDW gap edges. (a) STM image of V-intercalated monolayer VSe_2. (b) 2D conductance plot measured along the white line in (a). (c) Line profiles of the conductance plot in (b) along the red and purple horizontal lines, respectively. Spectroscopic conditions: V_b = -0.15 V, I_t = 200 pA, V_{mod} = 5 mV.

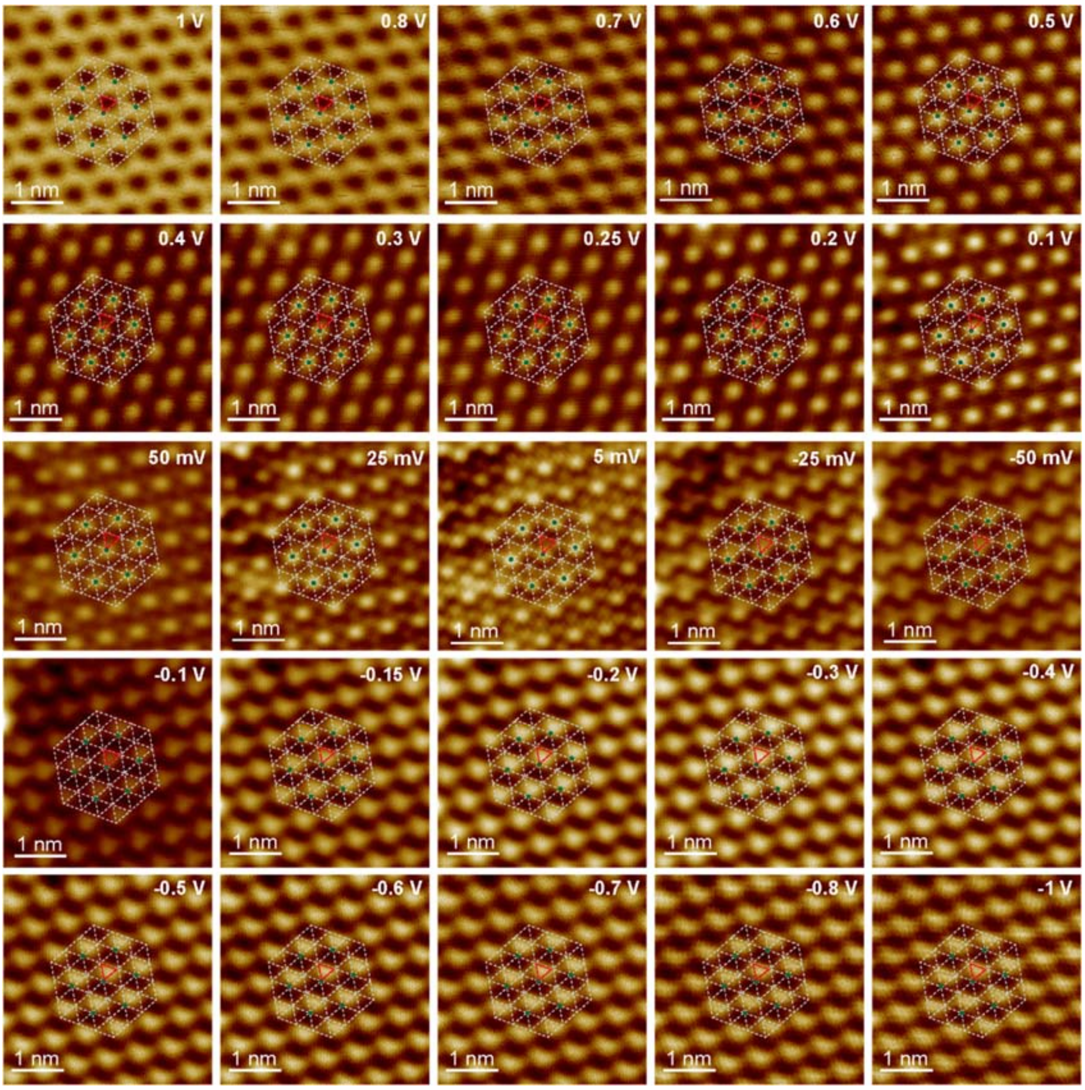

Fig. S6 Constant-current STM images of $\sqrt{3} \times \sqrt{3}$ VSe_2 at different bias voltages. The STM images show different patterns with changes in bias voltages (I_t = 10 pA). Image brightness is opposite under positive and negative bias. The red triangle marks a trimer, and the white dashed line and the green dot represent the 1×1 and the $\sqrt{3} \times \sqrt{3}$ period, respectively.

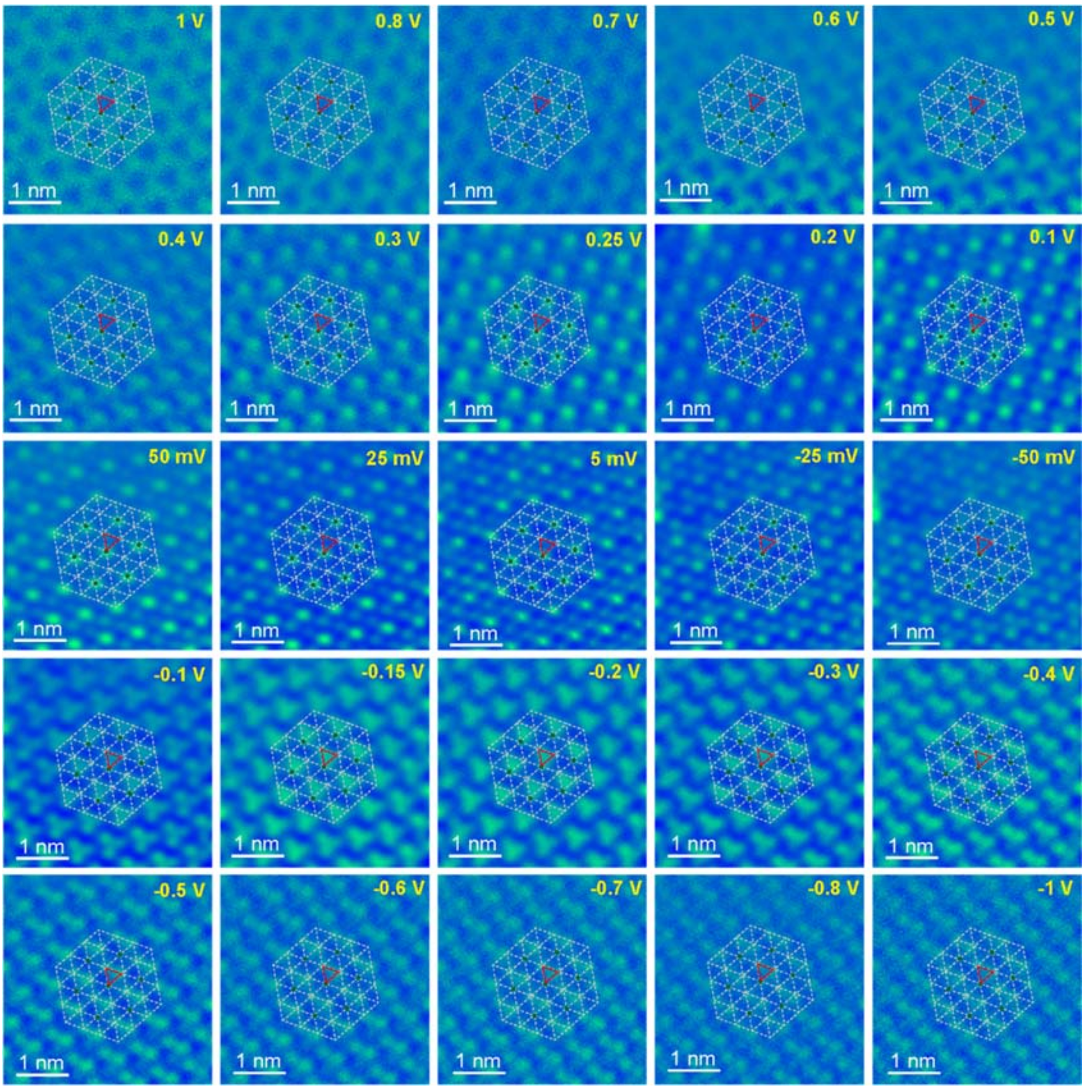

Fig. S7 Constant-height d*I*/d*V* mappings of $\sqrt{3} \times \sqrt{3}$ VSe_2 at different bias voltages. d*I*/d*V* mappings are very similar to STM images under the same bias. The evolution of the spatial distribution of the electronic states as the bias voltage changes can be clearly seen, as described in the main text. The red triangle marks a trimer, and the white dashed line and the green dot represent the 1×1 and the $\sqrt{3} \times \sqrt{3}$ period, respectively.

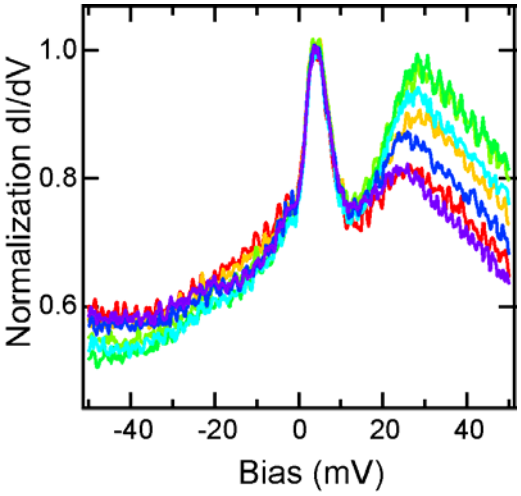

Fig. S8 Normalized d*I*/d*V* in a CDW period. In order to show more clearly the spatial homogeneity of the Kondo resonance, we selected the STS spectra from the line spectrum of Fig. 3(e) in a complete CDW period (marked by the pink dashed box), and the normalized spectra show that the Kondo resonance measured at different locations in space is identical.

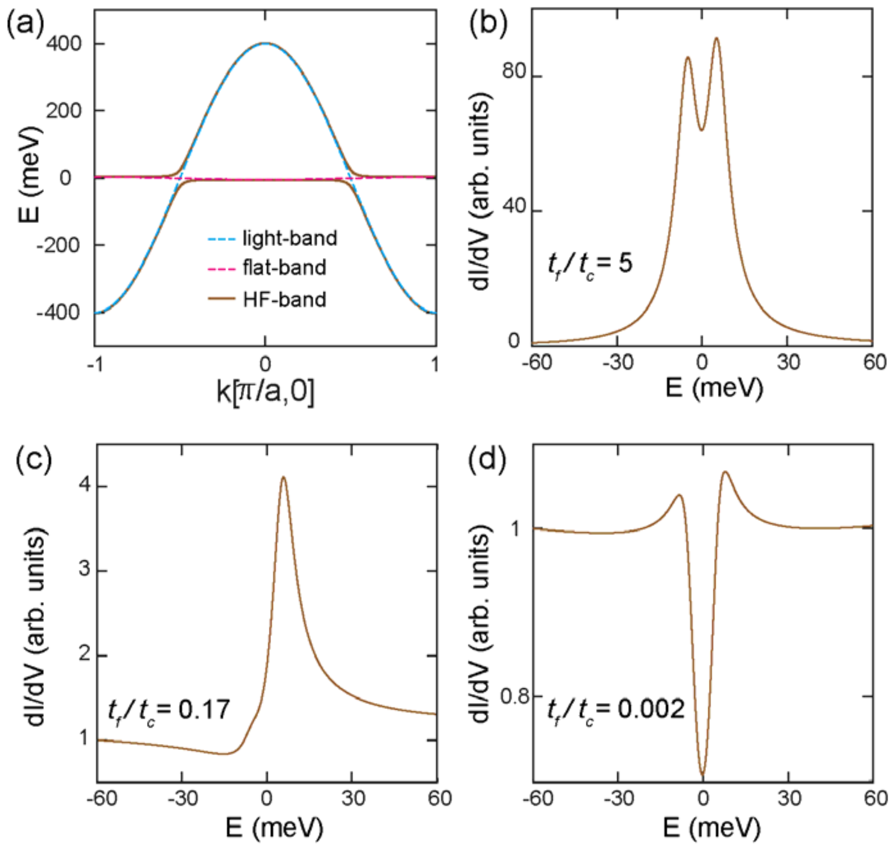

Fig. S9 Influence of tunneling path ratios on tunneling spectra. (a) Dispersion of the conduction light (dashed blue) and flat (dashed pink) electronic bands and the hybridized heavy fermion bands (solid brown) computed for $\mathrm{t} = 200\mathrm{meV}, \mu = 2\mathrm{t},\ \chi_0 = 0.01t, \varepsilon_0^f = 0.02\mathrm{t}, \nu = 0.12\mathrm{t}, \gamma_f = 0.022\mathrm{t}, \gamma_c = 0.03\mathrm{t},\ {\gamma_c}^{-1}$ and ${\gamma_f}^{-1}$ are the lifetimes of the light and flat electron states. (b-d) Differential conductance computed for the heavy fermion bands of (a), using different values of t_f/t_c.

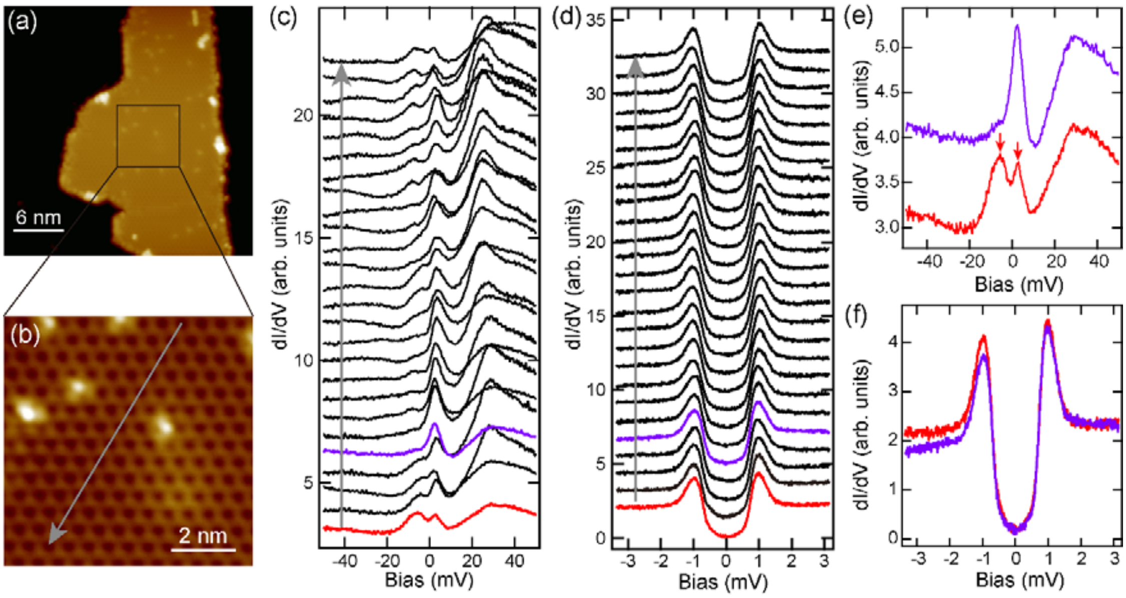

Fig. S10 Hybridization gap and superconducting proximity gap spectra in large VSe_2 island. (a) STM image of a typical monolayer VSe_2 island of large size (V_b = 1 V, I_t = 10 pA). (b) Zoom-in STM image of the rectangle area in (a) (V_b = 1 V, I_t = 10 pA). (c, d) dI/dV spectra measured along the gray line in (b), showing the hybridization gap feature (c) and the robust superconducting proximity gap (d), respectively. Spectroscopic conditions: V_b = -50 mV, I_t = 200 pA, V_{mod} = 0.5 mV for (c); V_b = 3.5 mV, I_t = 300 pA, V_{mod} = 0.03 mV for (d). Note that the spectra in (c) are measured at 6 T, suppressing the superconductivity. (e) Two typical dI/dV spectra extracted from the highlighted spectra in (c), exhibiting hybridization gap (red curve) and the single Kondo resonance peak (purple curve), respectively. Two red arrows mark the hybridization gap. (f) Two typical superconducting gap spectra extracted from the highlighted spectra in (d), showing same superconducting gap sizes but different spectral background slopes.

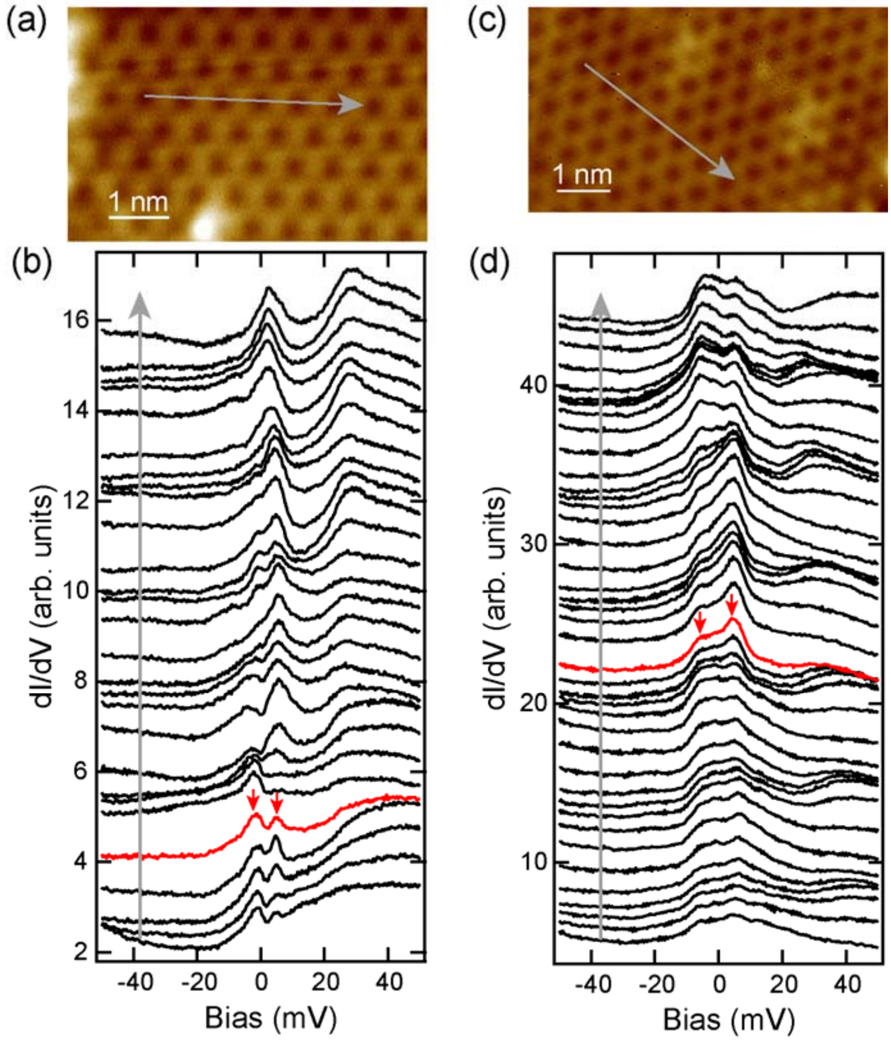

Fig. S11 Hybridization gap spectra on another two VSe_2 islands. (a, c) STM images of another two monolayer VSe_2 samples. Image conditions: V_b = 1 V, I_t = 10 pA for (a), V_b = 0.9 V, I_t = 30 pA for (c). (b, d) dI/dV spectra measured along the gray arrows in (a, c), respectively, exhibiting evident hybridization gap features. The hybridization gaps are marked with two arrows on two typical curves highlighted in red. Spectroscopic conditions: V_b = 50 mV, I_t = 200 pA, V_{mod} = 0.5 mV for (b); V_b = 50 mV, I_t = 300 pA, V_{mod} = 1 mV for (d).

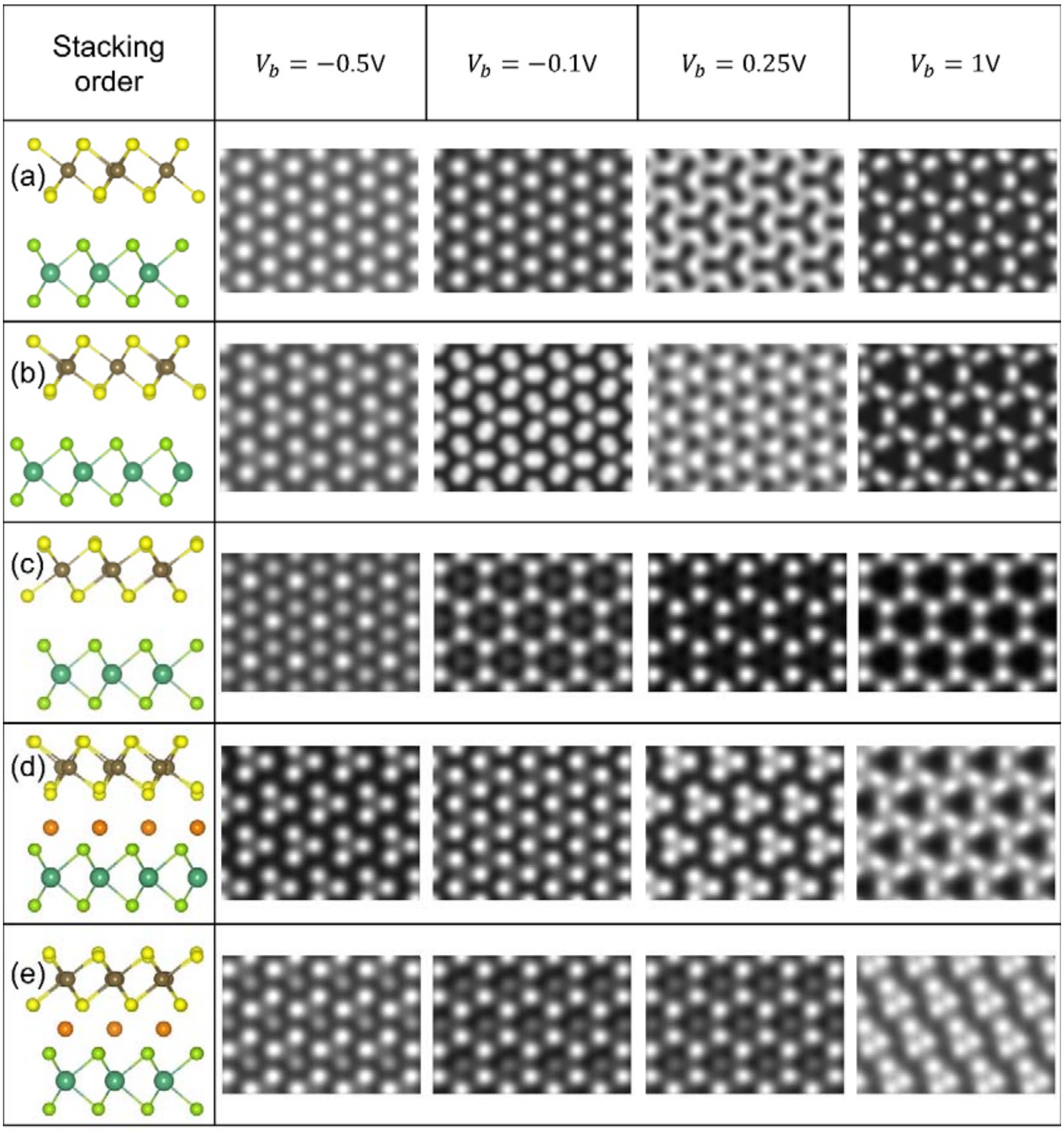

Fig. S12 Simulated dI/dV maps of different stacking structure with/without interstitial vanadium at typical bias voltage. (a)-(c) vdW structures without interstitial vanadium. (d)-(e) Same stacking order as (a) and (c) respectively but with interstitial vanadium. Interstitial vanadium atoms are illustrated with orange.

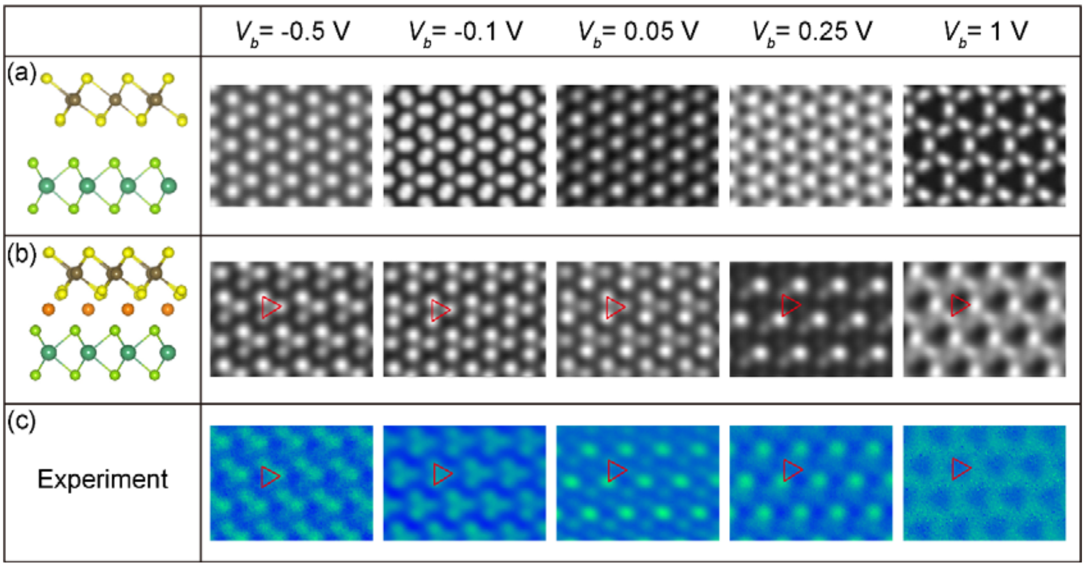

Fig. S13 Comparison between experimental d*I*/d*V* maps and DFT-simulated maps. (a) DFT-simulated d*I*/d*V* maps for vdW heterostructure without intercalated V atoms, showing three-fold symmetry of the $\sqrt{3} \times \sqrt{3}$ pattern. (b) DFT-simulated d*I*/d*V* maps for vdW heterostructure with intercalated V atoms, showing reduced symmetry and conforming to the experiments. (c) Experimental constant-height d*I*/d*V* maps at corresponding biases. The red triangles mark the trimer unit.

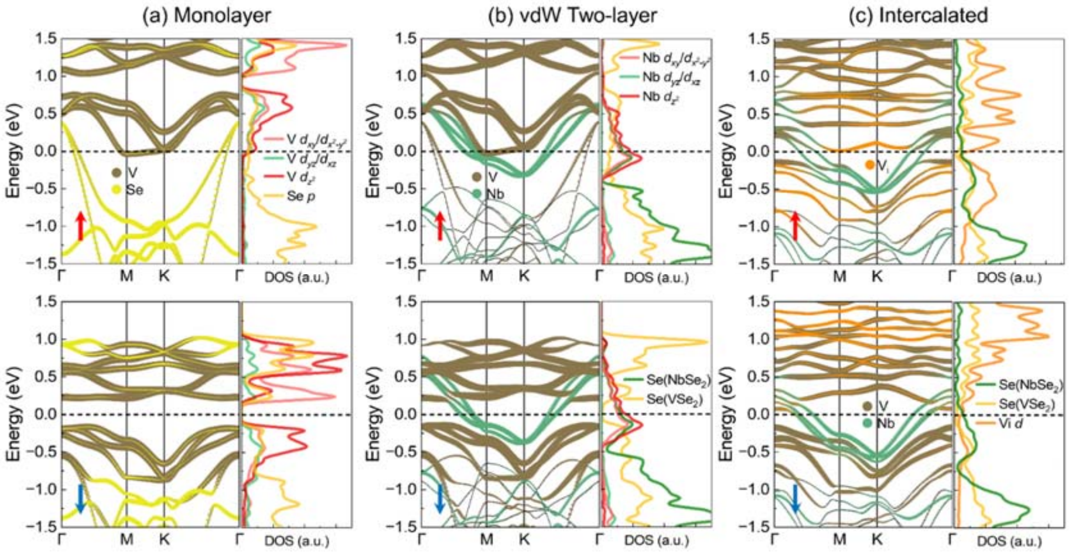

Fig. S14 Comparation of the electronic properties of between $\sqrt{3} \times \sqrt{3}$ monolayer VSe_2, $VSe_2/NbSe_2$ vdW two-layer and the V-intercalated $VSe_2/NbSe_2$ structure. Projected band and density of states of (a) monolayer VSe_2, (b) $VSe_2/NbSe_2$ vdW two-layer and (c) the V-intercalated structure. The red (blue) arrows represent spin-up (down) channel. The Fermi level is set to zero.

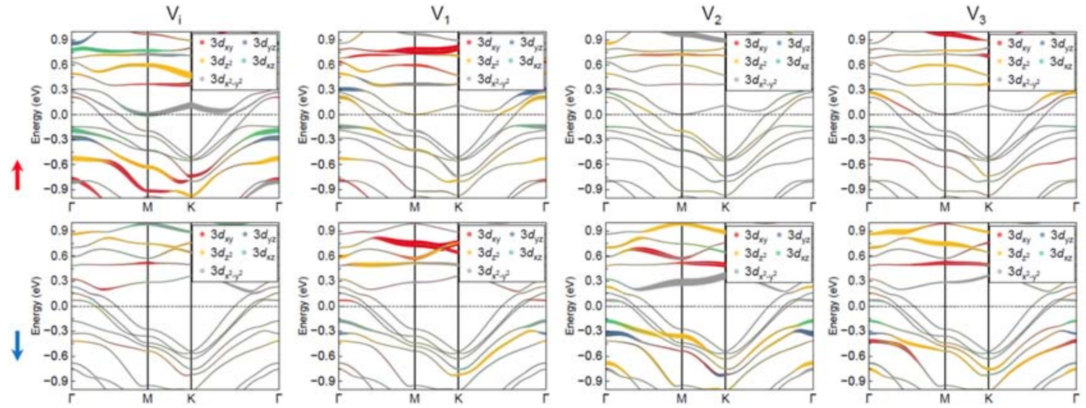

Fig. S15 3*d* orbital-projected band structure of V_i and V in the intercalated structure.

The labels of V atoms are defined in Fig. 1(d). The Fermi level is set to zero.

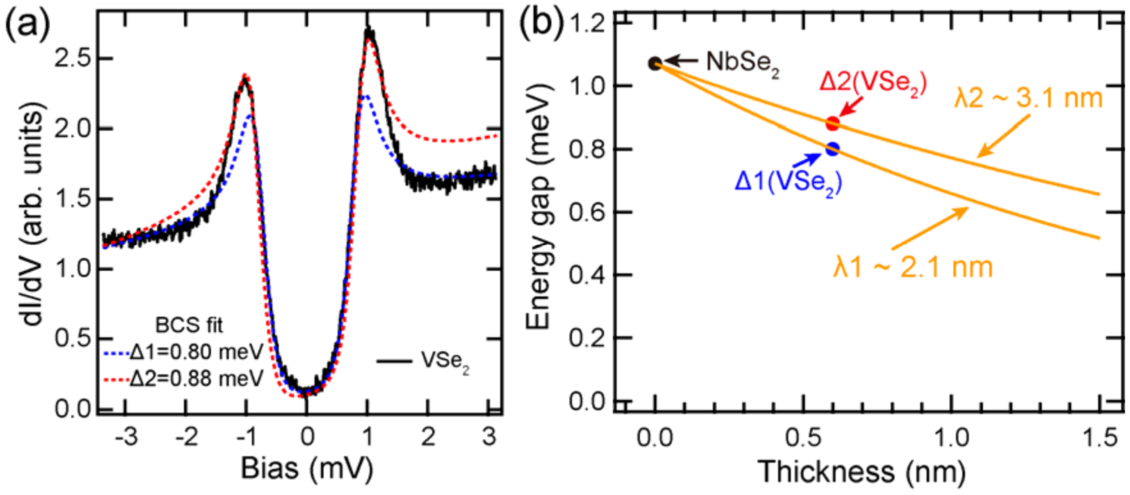

Fig. S16. Estimation of the decay length of the superconducting proximity effect. (a) Superconducting proximity gap spectrum of monolayer VSe_2 (black curve) and the energy gap Δ obtained by BCS fittings. Spectroscopic conditions: V_b = 3.5 mV, I_t = 200 pA, V_{mod} = 0.03 mV. Two fits are employed, with one by fitting the bottom of the superconducting gap to obtain Δ1 = 0.80 meV (blue curve) and the other by fitting the superconducting coherence peaks to obtain Δ2 = 0.88 meV (red curve). The former (latter) underestimates (overestimates) the superconducting gap, thus putting a lower (upper) bound to the gap size. Note that a linear background is added for the fitting. (b) The decay lengths of the superconducting proximity effect obtained from an exponential fitting to the superconducting gap Δ of bare $NbSe_2$ [Fig. 4(c)] and the intercalated monolayer VSe_2 [Fig. S16(a)].

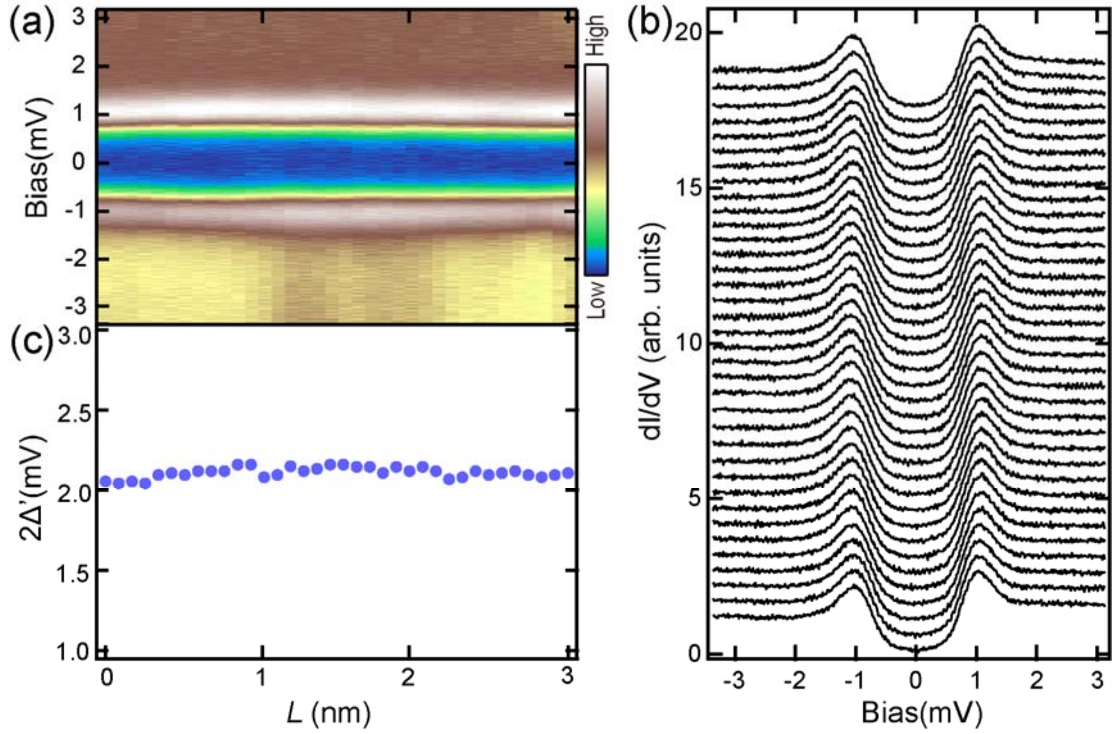

Fig. S17 Spatial distribution of SC gap at homogeneous regions. (a-b) 2D conductance plots and corresponding d*I*/d*V* spectra of the SC gap measured along the white line in Fig. 3(d). The spectra are vertically offset for clarity. Spectral conditions: V_b = 3.5 mV, I_t = 200 pA, V_{mod} = 0.03 mV. (c) Extracted the bias difference between a pair of coherence peaks 2Δ' of the d*I*/d*V* spectra shown in (b). Fig. R2. (a) Superconducting proximity gap spectrum of monolayer VSe_2 (black line) and the energy gap Δ obtained by BCS fittings. Spectroscopic conditions: V_b = 3.5 mV, I_t = 200 pA, V_{mod} = 0.05 mV. Two fits are employed, with one by fitting the bottom of the superconducting gap to obtain Δ1 = 0.80 meV(blue line) and the other by fitting the superconducting coherence peaks to obtain Δ2 = 0.88 meV(red line). Note that a linear background is added for the fitting. (b) The decay lengths of the superconducting proximity effect obtained from fitting the SC gap Δ of $NbSe_2$ [Fig. 4(c)] and the intercalated monolayer VSe_2 [Fig. R2(a)].

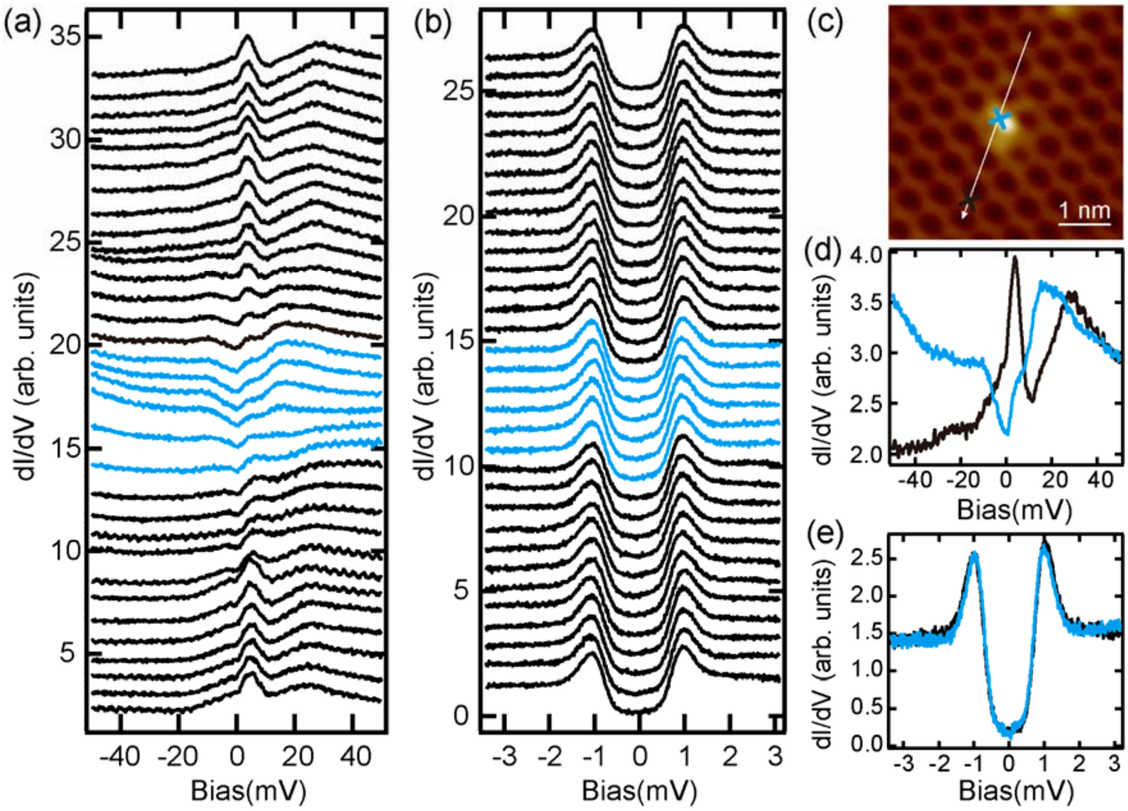

Fig. S18 Influence of an adsorbate on Kondo resonance peak and SC gap. (a) d*I*/d*V* spectra near the Fermi surface measured along the white line in (c). Spectral conditions: V_b = 50 mV, I_t = 200 pA, V_{mod} = 1 mV. (b) d*I*/d*V* spectra of the SC gap measured along the white line in (c). Spectral conditions: V_b = 3.5 mV, I_t = 200 pA, V_{mod} = 0.03 mV. The spectra are vertically offset for clarity. (c) STM image of VSe_2 surface containing an adsorbate (V_b = 1 V, I_t = 10 pA). (d, e) The d*I*/d*V* spectra on and away from the adsorbate whose spectral locations are marked with crosses in (c).

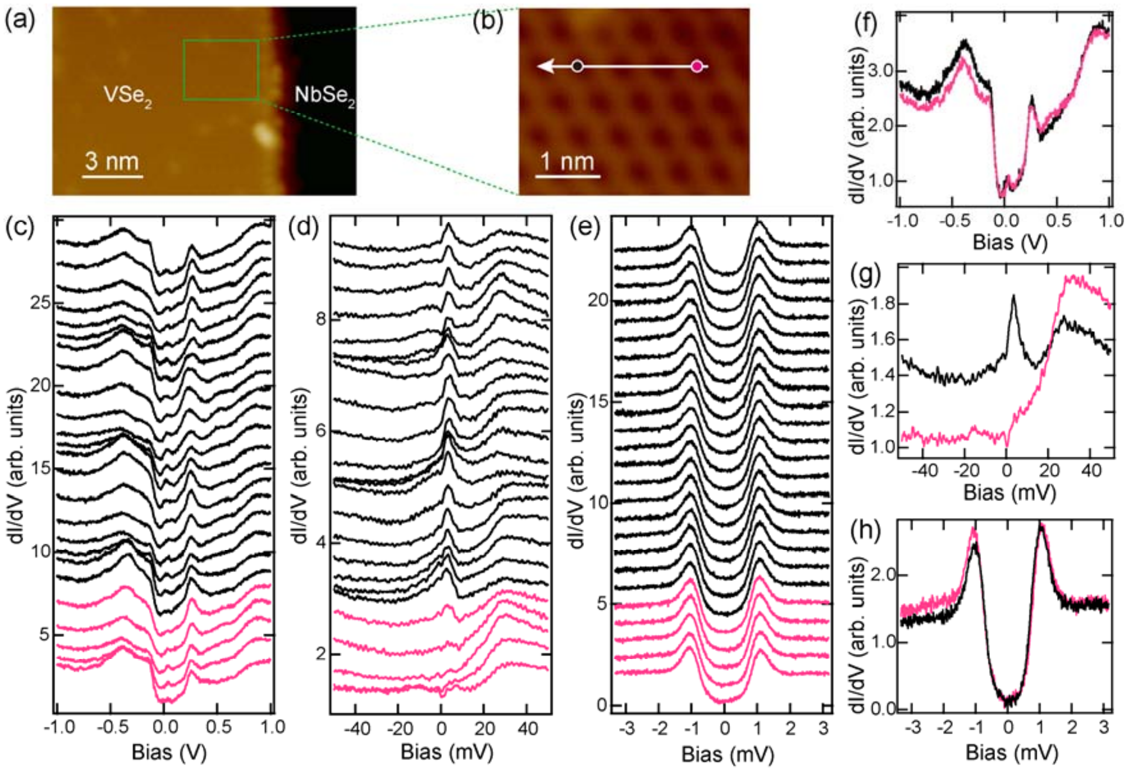

Fig. S19 Comparison of spectra at the inner and edge of VSe_2 island. (a) STM image (V_b = 1 V, I_t = 10 pA) of a monolayer VSe_2 island, whose zoom-in image (b) shows a regular CDW pattern close to the island edge. (c-e) dI/dV spectra measured along the white line in (b), showing uniform overall electronic structure (c), gradual disappearance of the Kondo resonance (d), and the robust proximity superconducting gap (e). Spectral conditions: V_b = 1 V, I_t = 150 pA, V_{mod} = 20 mV for (c); V_b = 50 mV, I_t = 200 pA, V_{mod} = 0.5 mV for (d); V_b = 3.5 mV, I_t = 200 pA, V_{mod} = 0.03 mV for (e). The spectra are vertically offset for clarity. (f-h) Selected dI/dV spectra at the locations marked with black and pink dots in (b), showing the comparison between the spectra of the inner and edge of the island.

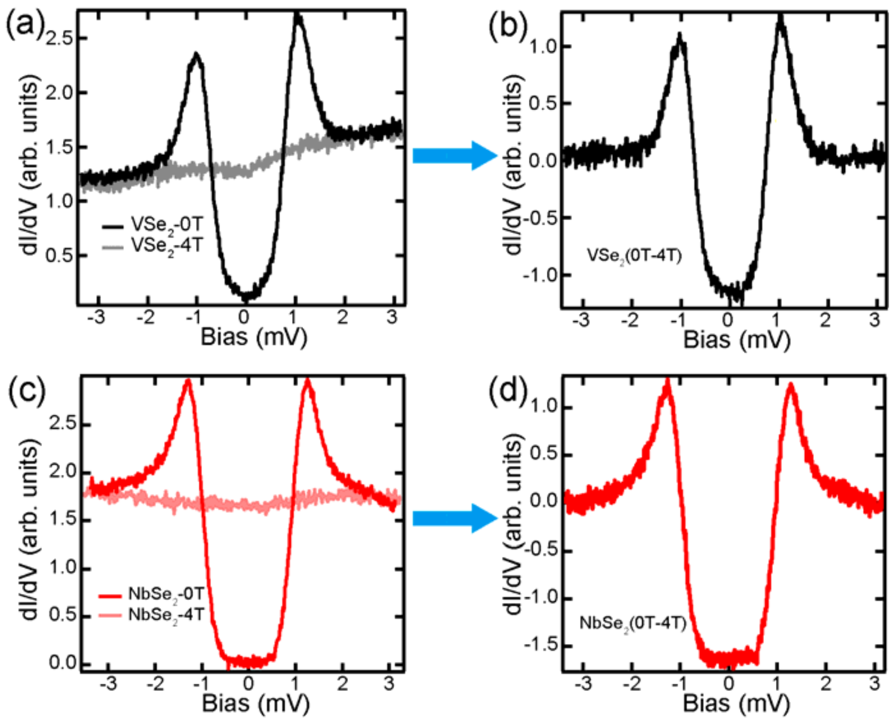

Fig. S20 Removing the tilted background of the SC gap of $\sqrt{3} \times \sqrt{3}$ VSe_2. (a) The black and gray lines are the dI/dV spectra of the SC gap detected at the same point with an applied magnetic field of 0T and 4T, respectively. It is obvious that the SC gap has been completely quenched at 4T, leaving only a sloping background. (b) By subtracting the two spectra in a, the SC energy gap of VSe_2 after background removal is obtained. (c-d) The same process applies to the SC gap of $NbSe_2$. Spectral conditions: V_b = 3.5 mV, I_t = 200 pA, V_{mod} = 0.03 mV.

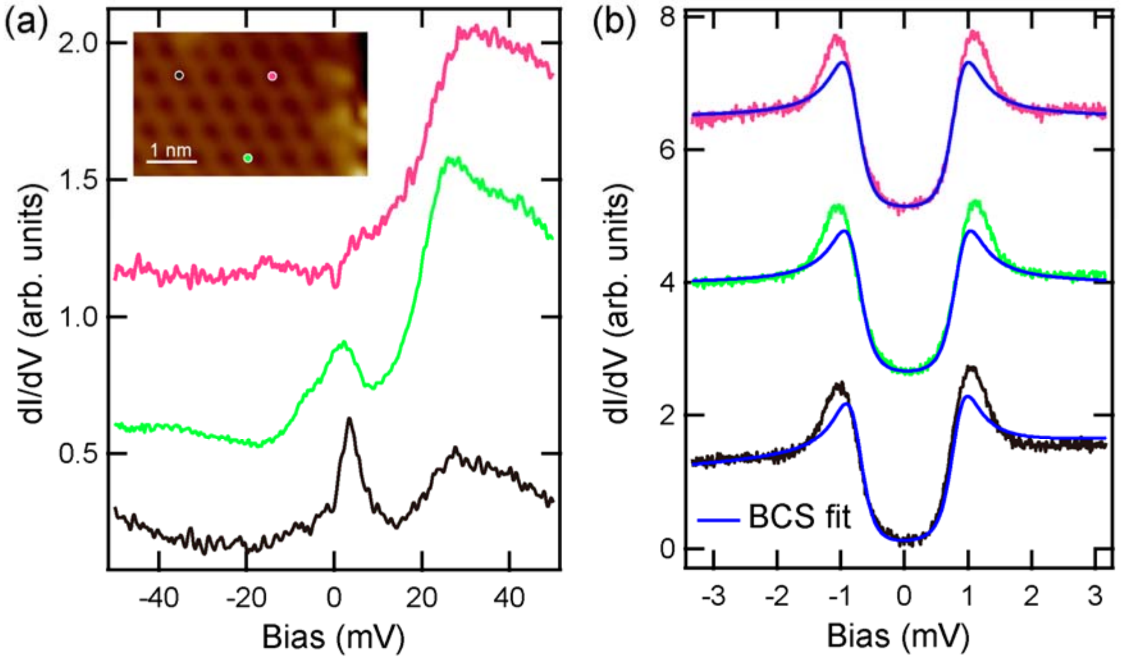

Fig. S21 Different shapes of Kondo lattice spectra and their superconducting proximity gaps. (a) dI/dV spectra of monolayer VSe_2 measured at different locations of the inset image (V_b = 1 V, I_t = 10 pA), showing variations of the Kondo lattice spectra near an island edge. Spectroscopic conditions: V_b = 50 mV, I_t = 200 pA, V_{mod} = 0.5 mV. (b) Superconducting gap spectrum measured at the same locations in the inset image of (a). Spectroscopic conditions: V_b = 3.5 mV, I_t = 200 pA, V_{mod} = 0.03 mV.

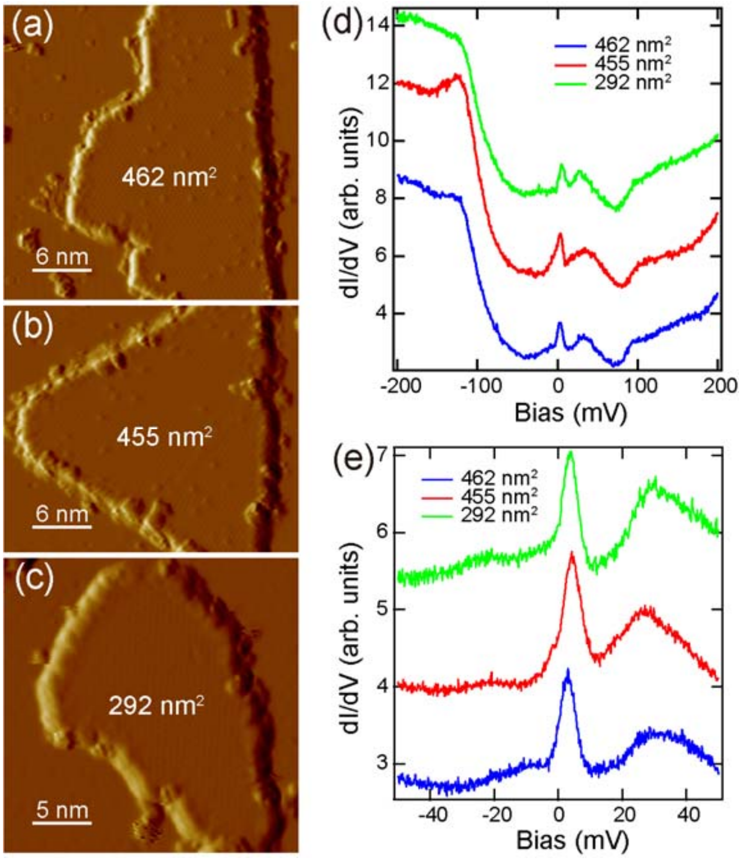

Fig. S22 Excluding quantum size effects in VSe_2 islands. (a-c) STM images of three different sizes of VSe_2 islands. (d, e) Small energy range spectra and Kondo resonance spectra on three islands. It can be seen that islands of different sizes exhibit the same spectra features and no effect of quantum size effects are observed. Spectroscopic conditions: (d) $V_b = 0.2$ V, $I_t = 200$ pA, $V_{mod} = 4$ mV; (e) $V_b = 50$ mV, $I_t = 200$ pA, $V_{mod} = 0.5$ mV.
