# Supplementary material for: Artificial superconducting Kondo lattice in a van der Waals heterostructure
Source: Nat Commun. 2024 Oct 11;15:8797. doi: 10.1038/s41467-024-53166-9 (PMC11470022; doi:10.1038/s41467-024-53166-9)
Supplement: Supplementary file 2 — Peer Review file [file 41467_2024_53166_MOESM2_ESM.pdf]

nature portfolio

Peer Review File

Artificial superconducting Kondo lattice in a van der Waals heterostructure

Corresponding Author: Professor Yingshuang Fu

This manuscript has been previously reviewed at another journal. This document only contains reviewer comments, rebuttal and decision letters for versions considered at Nature Communications.

Version 0:

Reviewer comments:

Reviewer #1

(Remarks to the Author)
Having reviewed the revised manuscript and the authors' responses, I am satisfied that they have adequately addressed my earlier concerns. I recommend the manuscript for publication in Nature Communications.

Reviewer #2

(Remarks to the Author)
I have read the revised manuscript and the authors' responses to the comments of reviewer 1 and 2. There were rather critical and important comments from the reviewer 2, which I believe were largely made clear by the extra data and arguments provided by the authors. I support the prompt publication of this work in Nature Communications.

Open Access This Peer Review File is licensed under a Creative Commons Attribution 4.0 International License, which permits use, sharing, adaptation, distribution and reproduction in any medium or format, as long as you give appropriate credit to the original author(s) and the source, provide a link to the Creative Commons license, and indicate if changes were

made.
In cases where reviewers are anonymous, credit should be given to 'Anonymous Referee' and the source.
The images or other third party material in this Peer Review File are included in the article’s Creative Commons license, unless indicated otherwise in a credit line to the material. If material is not included in the article’s Creative Commons license and your intended use is not permitted by statutory regulation or exceeds the permitted use, you will need to obtain permission directly from the copyright holder.
To view a copy of this license, visit https://creativecommons.org/licenses/by/4.0/
